# Multiscale Dissolution Simulation of Particles from a Tablet in Dissolution Apparatus by Coupling Discrete Element with Lattice Boltzmann Methods

**DOI:** 10.1007/s11095-026-04111-6

**Published:** 2026-05-20

**Authors:** Yue Li, Peng Hou, Lei Xing, Kinam Park, Tonglei Li

**Affiliations:** 1 Department of Industrial and Molecular Pharmaceutics, Purdue University, 575 Stadium Mall Dr., West Lafayette, IN 47907 USA; 2 School of Chemistry and Chemical Engineering, University of Surrey, Guildford, GU2 7XH UK

**Keywords:** Disintegration, Dissolution, Drug dissolution kinetics, LBM-DEM coupling, Particle dynamics, USP apparatus II

## Abstract

**Purpose:**

Dissolution kinetics of tablets is pivotal to therapeutic performance, quality assessment, and troubleshooting in manufacturing. We aimed to develop and validate a simulation framework to predict tablet dissolution kinetics under realistic hydrodynamic conditions, thereby supporting process development and quality control.

**Methods:**

We modeled the dissolution of drug particles packed within a tablet by coupling the lattice Boltzmann method (LBM) for fluid flow and transport with the discrete element method (DEM) for particle mechanics in USP Apparatus II. The framework was first validated against experimental measurements of single‑particle dissolution, then scaled to bulk-particle (tablet‑level) simulations to assess model extensibility.

**Results:**

The coupled LBM-DEM framework reproduced experimentally observed hydrodynamics and particle dynamics in a mechanically agitated dissolution device and scaled from single‑particle to tablet‑level simulations without sacrificing fidelity.

**Conclusions:**

This physics‑based framework enables the prediction of tablet dissolution under compendial hydrodynamic conditions and provides a foundation for incorporating additional particle‑mechanical phenomena (e.g., swelling and breakage) to fully model tablet disintegration and dissolution.

## Introduction

Thanks to patient compliance and the feasibility of mass production, tablet formulation is widely accepted in the pharmaceutical industry. The drug release kinetics of a tablet determines its bioavailability. Testing disintegration and dissolution kinetics allows the evaluation and optimization of the drug manufacturing process and enables the establishment of *in vivo*-*in vitro* correlation (IVIVC). Several compendial methods have been developed based on standard devices for the quality control of tablet production. As a widely used device, the United States Pharmacopeia (USP) Dissolution Apparatus II consists of a rotating paddle in a cylindrical vessel [1] and generally requires one tablet in a large amount of buffer solution (e.g., 900 mL). Under a constant stirring rate (e.g., 50 rpm) and temperature (37 °C), drug concentration is sampled at a particular position in the vessel at predetermined time points. Because of the hydrodynamic environment, it remains questionable whether reliable and representative measurements can be obtained by the current compendial method [2–4]. Some of the variability could be avoided by careful calibration with calibrator tablets [5]. However, in some situations, variability and inconsistency persist even after calibration [5–7]. This is usually due to the complexity of hydrodynamic profiles near the rotation paddle in a testing apparatus [8]. With its rotation at 50 rpm under standard conditions, the paddle’s tip is measured at 0.194 m s^−1^ with a Reynolds number as high as 4,939 [9], which suggests heterogeneous time-dependent flow dynamics in the stirred vessel.

Experimental studies are reported in the literature to understand the hydrodynamics within a dissolution device, such as the USP Apparatus II, and its impact on drug release [6, 10, 11]. The release of dye molecules from a tablet helped visualize the interplay between liquid flow and concentration distribution [2]. Laser-induced fluorescence (LIF) was utilized to gain quantitative information about tablet dissolution in the USP Apparatus II [7]. It was shown that by releasing fluorescent dye molecules from a tablet located at the bottom of a dissolution vessel, the mixing pattern and concentration distribution of drug particles or molecules with the dissolution media could be captured under the illumination of a planar laser. Laser Doppler velocimetry was utilized to measure liquid velocities at a selected location in a dissolution apparatus [12, 13]. The measurement could yield quantitative information about fluid hydrodynamics at isolated points. Still, obtaining a complete picture of the hydrodynamic field and concentration distribution with the current experimental means is difficult.

Conversely, fluid-dynamics computations and particle-disintegration simulations may provide a detailed understanding of tablet dissolution kinetics. Compared with experimental measurement, mathematical modeling is usually free of experimental errors due to the operation and the surrounding environment [14–16]. There has been an increase in computational studies on tablet disintegration in dissolution devices reported in the literature. Some implemented computational fluid dynamics (CFD) to simulate detailed hydrodynamic evolution in the USP Apparatus II [6, 8–10, 13, 17, 18]. Simulation results were compared with experimental data obtained by laser Doppler velocimetry (LDV) and particle image velocimetry (PIV) [7, 17]. CFD simulation can provide further insights into the disintegration dynamics of a tablet under mechanical flow. One study showed that the shear rate of liquid could dramatically vary along the bottom of the USP Apparatus II vessel, suggesting that the tablet's location can affect the testing results from the compendial method [8]. The finding is further confirmed by another simulation work [18]. Moreover, CFD simulation was also used to examine the impact of paddle agitation speeds on fluid strain rate distribution [13].

However, CFD alone cannot capture tablet formulation and the associated influence on the kinetics of tablet disintegration and dissolution. While the geometry of a tablet can be integrated into a CFD simulation and fixed in a dissolution vessel to examine hydrodynamic conditions at the tablet surface, it is difficult to thoroughly examine the interaction between the fluid phase and the solid tablet or particles, especially when drug particles undergo dissolution. In addition, by coupling CFD and direct numerical simulation (DNS) of drug particles, the dissolution process of the particle under hydrodynamic conditions was simulated [19, 20]. Nonetheless, it appears in this reported study that only one way of coupling from CFD to DEM was achieved [20, 21], ignoring any possible impact of particle movement on liquid flow. This would prevent particle behavior from being transferred to fluid dynamics, so this approach is not suitable for systems with a large number of particles. To achieve the two-way coupling of the interaction of particles and liquid, we resorted to using the lattice Boltzmann method (LBM) to compute the hydrodynamics of liquid and integrate DEM to simulate particle dynamics.

As an emerging CFD method, LBM has gained considerable attention [22–25]. Compared to traditional FEM- and FVM-based CFD methods, LBM is simpler to implement and better at handling complex boundaries and multiphase and multicomponent flows [26, 27]. Because of the nature of local dynamics, LBM can be easily implemented in parallel computation for large-scale simulations [28]. More importantly, the coupling between hydrodynamics and particle dynamics is more easily implemented using LBM. There are generally two primary coupling methods: the momentum exchange method (MEM) [29, 30] and the partially saturated cell method (PSM) [31]. In MEM, a particle is mapped into the fluid domain, and the occupied LBM lattice cell is treated as solid. Hydrodynamic forces on the particle surface can be derived by applying non-sliding boundary conditions on the particle surface. Conversely, a counter-acting force with the same magnitude acts on the fluid. In PSM, interaction forces between the fluid and a particle are evaluated based on the ratio of volume filled by the particle in the occupied lattice cell(s) and are utilized in Boltzmann kinetic equations. PSM is generally regarded as a more precise and flexible approach for simulating particle–fluid interactions, especially in scenarios involving intricate geometries and high particle densities, because it explicitly accounts for particle volume within each computational cell [32–34]. As a result, PSM, also called the immersed moving boundary method (IMBM), is used in our simulation platform.

Most existing CFD simulations of the drug dissolution process only model the velocity field in the USP Apparatus II [18, 35]. As a result, tablet dissolution is often simulated by calculating dissolution kinetics on an idealized tablet surface [18]. Other studies first simulate the velocity field and then use it to calculate the concentration field [19, 20]. This workflow is not easily scalable, and the coupling is typically unidirectional, meaning the effects of particle movement and local dissolution on the surrounding hydrodynamics are ignored. Additionally, related work on pharmaceutical tableting has shown that realistic compaction behavior may require more detailed particle-mechanical descriptions than pairwise overlap alone. For example, combined finite-discrete element formulations have been used to simulate pharmaceutical powder tableting [64], and multi-contact DEM has been used for high-density tablet compaction [65]. More recent research from the Kwade group has further highlighted formulation- and structure-dependent behavior by linking excipient composition and porosity reduction to properties like compressibility, compactibility, and tabletability in complex granules [67]. They also extended the CFD-DEM approach to bonded and deformable, impermeable particle structures through a bond-volume-aware void-fraction treatment [68].

Meanwhile, Feng and colleagues have developed a high-resolution DEM-LBM-IMB method for particle-resolved coupling problems, including the reassessment of empirical DEM-CFD coupling laws through particle-resolved simulations [69] and grain-resolved heat-particle–fluid coupling with explicit lattice-unit conversion and benchmark validation [70]. Watano and colleagues have also demonstrated that compression speed, residual stress, crack formation, and capping can significantly influence the final tablet structure, using DPC-Perzyna/FEM and X-ray CT analyses to link compaction conditions with internal damage and failure signatures [66, 71, 72]. Collectively, these studies highlight that compaction history, excipient-dependent microstructure, and internal damage can materially impact the initial state presented to a dissolution model. They therefore motivate the specific focus of this work: our goal is not to replicate all aspects of the physics involved in tablet formation but to establish a two-way coupled LBM-DEM dissolution framework that captures fluid flow, particle motion, and dissolution in USP Apparatus II, using a simplified DEM-generated tablet as the starting point.

## Model Development

A multiscale simulation approach was implemented to study the disintegration dynamics of tablets in a dissolution device, USP Apparatus II. Our research goal is to understand and, eventually, predict how formulation factors, manufacturing process, and tablet microstructure jointly influence the drug release kinetics of a tablet under various hydrodynamic conditions of different paddle speeds. This work focused on developing a fully coupled LBM-DEM framework for future particle dissolution studies.

### Lattice Boltzmann Method (LBM) for Fluid Hydrodynamics

In our model, the lattice Boltzmann method is coupled with the discrete element method to study particle–fluid interactions.

This species conservation can be modeled by a convection–diffusion scheme described below:

1 $$\frac{\delta c}{\delta t}=\nabla \cdot \left(\mathrm{D}\nabla \mathrm{c}\right)-\nabla \cdot \left(\mathbf{v}\mathrm{c}\right)+R$$

where c is the drug concentration, t is time, D is the molecular diffusion coefficient, ***v*** is the local fluid velocity, and R is the source term associated with dissolution. In the discrete LBM formulation in Eq. 2, the subscript α denotes the α-th lattice direction, eα is the discrete lattice velocity vector along that direction, and δt is the numerical time step.

Considering the challenge of convergence and high computational load of traditional numerical approaches based on finite element method (FEM) and finite volume method (FVM), the convection–diffusion equation (Eq. 1.) is solved with the lattice Boltzmann method (LBM) by implementing two lattice equations as shown in Eq. 2 and Eq. 7 [36–38]. The function *f* (**x**,t) governs the hydrodynamic conditions, while the function *g(****x****,t)* governs the concentration distribution. The probability of finding a particle at location *x* at time *t* is equal to *f (****x****,t)* and *g(****x****,t)*. By passing the velocity field in *f* to *g*, we can simulate the impact of hydrodynamic conditions on mass concentration distribution.

2 $${f}_{\alpha }\left({\boldsymbol{x}}+{e}_{\alpha }\delta t,t+\delta t\right)-{f}_{\alpha }\left({\boldsymbol{x}},t\right)=-\frac{1}{\tau }{[{f}_{\alpha }\left({\boldsymbol{x}},t\right)- {f}_{\alpha }}^{eq}({\boldsymbol{x}},t)]$$

The viscosity of fluid *v* is related to the dimensionless relaxation time *τ*, as shown in Eq. 3, where *c*_*s*_ is the lattice sound speed.

3 $$v={\mathrm{c}}_{\mathrm{s}}^{2}\left(\tau -\frac{1}{2}\right)\delta t$$

The local equilibrium distribution function ${{f}_{\alpha }}^{eq}({\boldsymbol{x}},t)$ could be expressed as below:

4 $${{f}_{\alpha }}^{eq}=\uprho {\mathrm{w}}_{\alpha }\left[1+\frac{1}{2}\frac{{\boldsymbol{u}}\cdot {{\boldsymbol{c}}}_{{\boldsymbol{\alpha}}}}{{c}_{s}^{2}}+{\left(\frac{{\boldsymbol{u}}\cdot {{\boldsymbol{c}}}_{{\boldsymbol{\alpha}}}}{{c}_{s}^{2}}\right)}^{2}-\frac{{u}^{2}}{{2c}_{s}^{2}}\right]$$

where the fluid density *ρ* and velocity ***u*** could be calculated as below:

5 $$\rho ={\sum}_{\alpha =0}^{q}{f}_{\alpha } , \rho {\boldsymbol{u}}= {\sum}_{\alpha =0}^{q}{f}_{\alpha }{{\boldsymbol{c}}}_{{\boldsymbol{\alpha}}}$$

We can represent the right side of Eq. 2 as Ω:

6 $$\Omega =-\frac{1}{\tau }{[{f}_{\alpha }\left({\boldsymbol{x}},t\right)- {f}_{\alpha }}^{eq}({\boldsymbol{x}},t)]$$

The LBM equation for concentration distribution is:

7 $${g}_{\alpha }\left({\boldsymbol{x}}+{e}_{\alpha }\delta t,t+\delta t\right)-{g}_{\alpha }\left({\boldsymbol{x}},t\right)=-\frac{1}{{\tau }{\prime}}{[{g}_{\alpha }\left({\boldsymbol{x}},t\right)- {g}_{\alpha }}^{eq}({\boldsymbol{x}},t)]$$

Similarly, the local equilibrium distribution function ${{g}_{\alpha }}^{eq}({\boldsymbol{x}},t)$ could be expressed as below:

8 $${{g}_{\alpha }}^{eq}={\mathrm{Cw}}_{\alpha }\left[1+\frac{1}{2}\frac{{\boldsymbol{u}}\cdot {{\boldsymbol{c}}}_{{\boldsymbol{\alpha}}}}{{c}_{s}^{2}}+{\left(\frac{{\boldsymbol{u}}\cdot {{\boldsymbol{c}}}_{{\boldsymbol{\alpha}}}}{{c}_{s}^{2}}\right)}^{2}-\frac{{u}^{2}}{{2c}_{s}^{2}}\right]$$

The concentration or density of drug *C* could be described as:

9 $$C={\sum}_{\alpha =0}^{q}{g}_{\alpha }$$

The diffusion coefficient *D* could be described as:

10 $$D={\mathrm{c}}_{\mathrm{s}}^{2}\left({\tau }{\prime}-\frac{1}{2}\right)\delta t$$

In Eq. 10, τ′ denotes the relaxation time used for the convection–diffusion lattice, to distinguish it from the hydrodynamic relaxation time τ in Eq. 3. The two equations have the same algebraic form because both viscosity and diffusivity are recovered from BGK-type relaxation, but τ and τ′ are calibrated for different transport processes. Equation 3 links τ to momentum transport, whereas Eq. 10 links τ′ to species transport.

For the D3Q19 and D3Q7 models, the discrete velocity vectors ${{\boldsymbol{c}}}_{{\boldsymbol{\alpha}}}$ and weighting factors *w*_*α*_ on each direction could be shown as in Eq. 11 and Eq. 12, respectively. In our method, the D3Q19 model is used for velocity simulation, and the D3Q7 model is used for concentration simulation [39, 40].

11 $$\left\{{{\boldsymbol{c}}}_{{\boldsymbol{\alpha}}},{w}_{\alpha }\right\}=\left\{\begin{array}{c}c\left[\left(\mathrm{0,0},0\right)\right],\frac{1}{3} \alpha =0 \\ \begin{array}{c} \\ c\left[\left(\pm \mathrm{1,0},0\right),\left(0,\pm \mathrm{1,0}\right),\left(\mathrm{0,0},\pm 1\right)\right],\frac{1}{18} \alpha =\mathrm{1,2},3,\dots ,6\end{array} \\ \begin{array}{c} \\ c\left[\left(\pm 1,\pm \mathrm{1,0}\right),\left(\pm \mathrm{1,0},\pm 1\right),\left(0,\pm 1,\pm 1\right)\right],\frac{1}{36} \alpha =\mathrm{7,8},9,\dots ,18\end{array}\end{array}\right.$$

12 $$\left\{{{\boldsymbol{c}}}_{{\boldsymbol{\alpha}}},{w}_{\alpha }\right\}=\left\{\begin{array}{c}c\left[\left(\mathrm{0,0},0\right)\right],\frac{1}{4} \alpha =0 \\ c\left[\left(\pm \mathrm{1,0},0\right),\left(0,\pm \mathrm{1,0}\right),\left(\mathrm{0,0},\pm 1\right)\right],\frac{1}{8} \alpha =\mathrm{1,2},3,\dots ,6.\end{array}\right.$$

To simulate the situation where a solid particle dissolves into fluid, we use the source term *C*_*w*_ to describe the change of drug concentration due to particle dissolution.

13 $${g}_{\alpha }\left({\boldsymbol{x}}+{{\boldsymbol{c}}}_{{\boldsymbol{\alpha}}}\delta t,t+\delta t\right)={g}_{\alpha }^{+}\left({\boldsymbol{x}},t\right)+2{w}_{\alpha }{C}_{w}$$

To simulate the hydrodynamic interaction between the fluid and solid particles, we implement the immersed moving boundary method [31]. This is done by replacing the right side of Eq. 2 with another form:

14 $$\Omega = \frac{1}{\tau }{(1-B)(f}^{eq}-f)+B{\Omega }^{S}$$

15 $$B=\frac{\varepsilon (\tau -0.5)}{\left(1-\varepsilon \right)+(\tau -0.5)}$$

where ε is the ratio of the solid particle in the current lattice node.

16 $${\Omega }^{S}={f}_{-}\left({\boldsymbol{x}},t\right)-f\left({\boldsymbol{x}},t\right)+{f}^{eq}\left(\rho ,{{\boldsymbol{U}}}_{{\boldsymbol{s}}}\right)-{f}_{-}^{eq}(\rho ,{\boldsymbol{u}})$$

In Eq. 16, the term ***U***_*S*_ represents the velocity of the solid node. Further,${f}_{-}\left({\boldsymbol{x}},t\right)$ is the bounce back form of $f\left({\boldsymbol{x}},t\right)$.

### Discrete Element Method (DEM) for Particle Trajectory

We model tablet particles as solid particles in DEM. Each particle is explicitly modeled and treated as the fundamental unit. A particle (e.g., API) may dissolve in liquid, decreasing particle size and releasing molecules to the environment. A particle (e.g., excipient) may take up water from the liquid environment, swell, and exert forces on neighboring particles (in the tablet). To model the drug and excipient particles in a tablet, we must consider the interaction or bonding force between them.

**Fig. 1:**
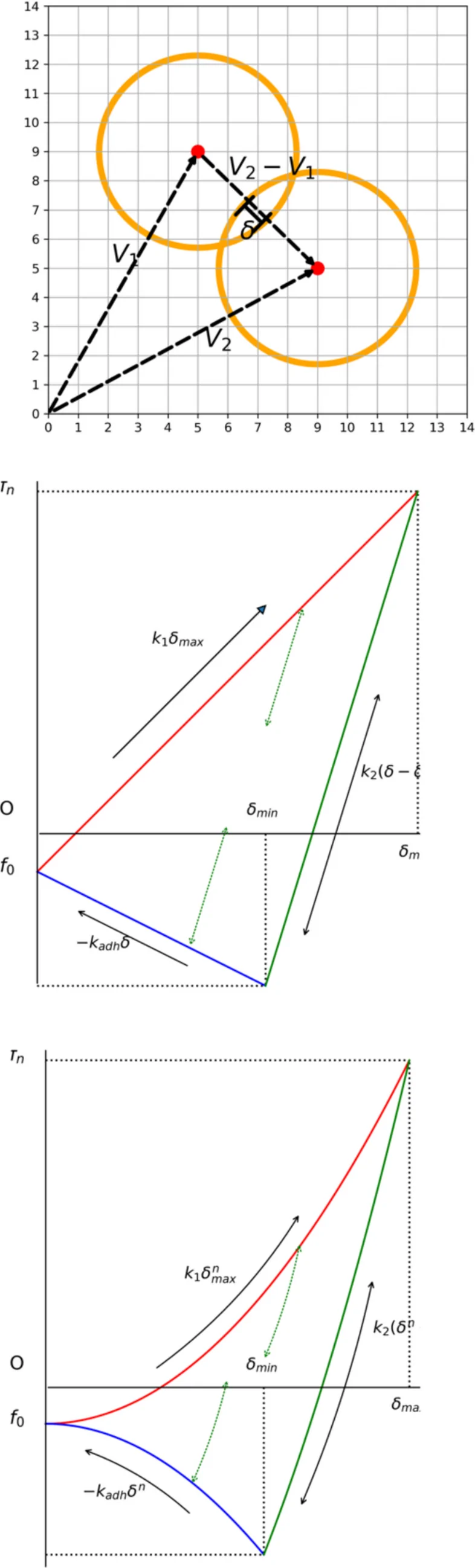
(Top) Geometric definition of particle overlap, δ, between two contacting particles. (Middle) Linear elasto-plastic-adhesive force–displacement response. (Bottom) Nonlinear elasto-plastic-adhesive force–displacement response.

As two solid particles approach each other, there is an attractive force due to the van der Waals interaction (and possible electrostatic interaction as well, if the particles bear surface charges). As they continue to get closer, especially during the tablet compression stage, the particles undergo deformation; firstly, the recoverable, elastic, and then (partially) plastic deformation. Such deformation requires energy, indicating the repulsive nature of a particle when compressed. As shown in Fig. 1, the first figure conveys an overlapping δ between two particles and is assumed to represent the elastic deformation, suggesting recovery of the particle, which Hooke’s law can describe. A few models have been used to describe interparticle interactions in DEM simulations. One such model is the Edinburgh Elasto–Plastic Adhesion (EEPA) Model, proposed by Thakur et al. in 2014 [41]. As shown in Fig. 1, the second and third figures model the load, unloading, reloading, and adhesive phases of particle–particle interactions. The loading stiffness parameter k_1_ governs the loading process; once unloaded or reloaded, the contact force will follow the unload/reloading stiffness parameter *k*_*2*_. If the unloading process continues, the contact force will switch to negative, which becomes an adhesive force until it reaches the maximum − *k*_*adh*_*δ*^*n*^_*min*_ + *f*_*0*_. If unloading continues, the adhesive force will decrease until it reaches a constant adhesive strength *f*_*0*_.

17 $${{\boldsymbol{f}}}_{{\boldsymbol{n}}}=({f}_{hys}+{f}_{nd}){\boldsymbol{u}}$$

18 $${f}_{hys} =\left\{\begin{array}{c}{f}_{0}+{k}_{1}{\delta }^{n} { k}_{1}{\delta }^{n} < {k}_{2}{(\delta }^{n}-{\delta}_{p}^{n}) \\ {f}_{0}+{k}_{2}{(\delta }^{n}-{\delta}_{p}^{n}) { k}_{adh}{\delta }^{n}<{k}_{2}{(\delta }^{n}-{\delta}_{p}^{n}) < {k}_{1}{\delta }^{n} \\ {f}_{0}-{k}_{adh}{\delta }^{n}{ k}_{2}{(\delta }^{n}-{\delta}_{p}^{n}) <{k}_{adh}{\delta }^{n}\end{array}\right.$$

19 $${f}_{nd}={\beta}_{n} {v}_{n}$$

where *ν*_*n*_ is the magnitude of the relative normal velocity, and *β*_*n*_ is the normal dashpot coefficient.

In a similar approach of using DEM to simulate the tableting process, when particles are pushed against each other, the same elastic repulsion model is used reciprocally to describe their attraction when pulled apart [42].

In the EEPA model, the adhesive force is considered the combination of several different forces, such as *van der Waals* forces, sintering and chemical bonding, electrostatic forces, mechanical forces, etc.

Our model employs a straightforward EEPA model to simulate particle interactions. Since the relative movement between particles is minimal, damping for *f*_*nd*_ is ignored in our model. The current tablet-compaction step is deliberately simplified and is used to create a mechanically plausible packed bed that serves as the initial microstructure for dissolution, rather than to replicate the full compaction history of a formulated tablet.

In the current setup, particles are loaded into a cylindrical die and compressed until a desired compact shape and average interparticle overlap are achieved. Therefore, the main variables related to compaction include particle size, particle count, die shape, punch displacement, contact-law parameters, and the resulting overlap distribution. The tablet solid fraction is not set as a separate input; instead, it results from the DEM packing and compression process and can be adjusted indirectly by changing the initial fill, particle properties, and the end point of compression.

The loading stiffness, unloading stiffness, adhesion stiffness, adhesion exponent, and overlap exponent were derived from the EEPA literature and then adjusted within physically reasonable limits to ensure numerically stable compaction and dissolution calculations. We acknowledge that this calibration is not yet a comprehensive material-specific identification process. Therefore, the manuscript now clearly states these parameters as literature-informed and stability-tuned rather than as strictly measured material constants.

A more rigorous calibration approach, such as direct particle-scale measurements, bulk compression-profile matching, or sensitivity analysis over the EEPA parameter set, would enhance the mechanical realism of the tablet-generation step. This is especially relevant when compared to more advanced multi-contact compaction frameworks. Therefore, we consider the current tablet-level simulations as a demonstration of framework extensibility rather than a fully validated predictive compaction model.

### Mass Transfer of DEM-simulated Particles to LBM-simulated Fluid

On the LBM side, we use the source term *C*_*w*_ as the mass flux in Eq. 13 to treat the mass transfer from the DEM-simulated solid particle to the LBM-simulated fluid field. On the DEM particle side, the drug dissolved from solid particles is simulated with the Noyes–Whitney equation, as shown below [43].

20 $$\frac{dW}{dt}=-\mathrm{K}\mathrm{A}\left({\mathrm{C}}_{\mathrm{s}}-\mathrm{C}\right)$$

where *A* is the surface of solid particles on the solid/liquid interface (m^2^), *K* is the dissolution constant (m/s), *C* is the current concentration, *C*_*s*_ is the saturated concentration (kg/m^3^), *W* is the weight of the particle (kg), and *t* is time (s).

Thus, in our simulation, the dissolution and convection–diffusion processes are considered two sequential steps. For each timestep, the source term on the unit lattice cell covered with the DEM particle could be expressed as below, where δt is the physical timestep, and δx is the unit physical length of each lattice node.

21 $${C}_{w}=-\frac{\delta t}{\delta x}\mathrm{K}\left({\mathrm{C}}_{\mathrm{s}}-\mathrm{C}\right)$$

This equation connects the DEM particle to the LBM fluid-mass transfer.

We calculate the mass transfer from the particle’s surface to the fluid to update the particle's radius owing to dissolution. The equation is described below:

22 $$\frac{d}{dt}\left({\rho}_{s}\frac{4\pi }{3}{\left({r}^{i}\right)}^{3}\right)=-{\int}_{{A}^{i}}\xi dA$$

where ξ is the mass flux (mass dissolved in per unit time at per unit lattice cell), *ρ*_*s*_ is the density of particle, and *r*_*i*_ is the radius of *i—th* particle in the DEM model.

Currently, in our model, as the particle dissolves and its radius becomes smaller than the lattice unit, we still use the immersed moving boundary method to handle the solid–fluid interaction. This might lead to a more significant error in calculating the interaction force. Further improvement could be implemented to recalibrate the interpolation function (Eq. 15).

### Force Interaction Between DEM-simulated Particles and LBM-simulated Fluid

The lattice Boltzmann method (LBM) has been developed for computing hydrodynamics since the 1970s [44–47]. Various review articles have been published on the methodology and its application [22, 26, 27, 48]. In brief, LBM treats fluid flow in a discretized lattice using particle or density distribution functions, from which the fluid velocity and pressure can be derived. The Boltzmann equation shown below highlights the essence of LBM, where Ω is the so-called collision operator that epitomizes the driving force(s) causing the change in LBM’s distribution function, *f*, over time.

23 $$\frac{\partial f}{\partial \mathrm{t}}+\mathrm{c}\bullet \nabla f=\Omega$$

In this equation, *c* is the lattice velocity. The collision term is generally unknown, but several empirical forms have been proposed, including the BGK model, developed by Bhatnagar, Gross, and Krook in 1954 [49], which is shown below:

24 $${\Omega =\omega (f}^{eq}-f)= \frac{1}{\tau }{(f}^{eq}-f)$$

In this equation, *ω* is the collision frequency and τ is the relaxation factor. With the BGK simplification, the Boltzmann equation becomes [22, 26, 50]:

25 $${f}_{\alpha }\left({\boldsymbol{x}}+{e}_{\alpha }\delta t,t+\delta t\right)-{f}_{\alpha }\left({\boldsymbol{x}},t\right)=-\frac{1}{\tau }{[{f}_{\alpha }\left({\boldsymbol{x}},t\right)- f}^{eq}({\boldsymbol{x}},t)]$$

The kinematic viscosity of the fluid is:

26 $${v}_{k}={\mathrm{c}}_{\mathrm{s}}^{2}\left(\tau -\frac{1}{2}\right)\delta t$$

where ${f}_{\alpha }$*(****x****,t)* is the density distribution function along *α* discretization direction of a lattice point ***x*** at time *t*.; *e*_*a*_ is the liquid velocity along *α* direction. The left side of the equation, also called “streaming,” describes the change in distribution function resulting from the exchange of momentum between neighboring lattice nodes, for example, due to underlying bulk advection and molecular diffusion. The dynamics of a liquid system are driven by the equilibrium distribution function, ${f}_{\alpha }^{eq}$*(****x****,t)*. Various schemes of simulation lattice and equilibrium distribution function have been developed and are widely used [51].

**Fig. 2:**
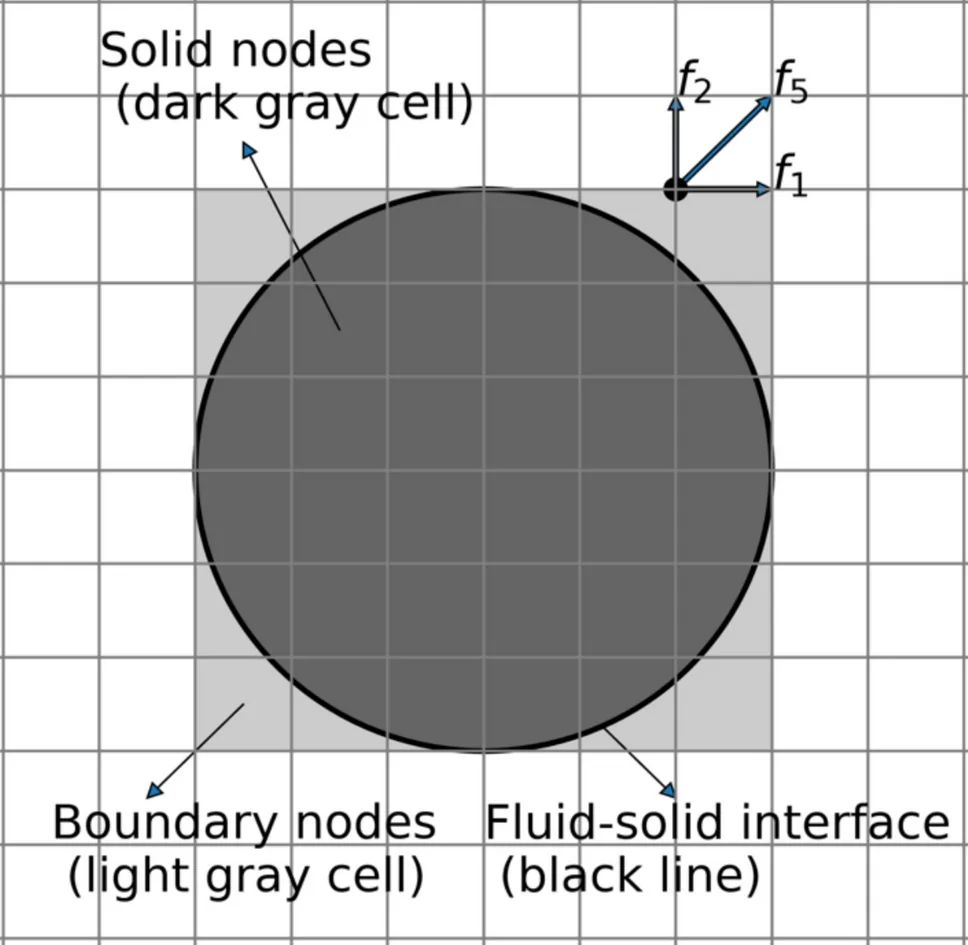
Solid particle (dark gray cell) in fluid (blank cell). The ratio of the solid part in a lattice cell is calculated based on the area of the dark grey part in a cell. The dark gray cells indicate the solid nodes, the light gray cells indicate the boundary nodes, and the blank white cells indicate the fluid nodes.

Solid particles are treated individually. The interaction between fluid and a solid particle is handled by the immersed moving boundary method [31]. At the fluid–solid interface (Fig. 2), local solid and fluid velocities are kept the same by adding additional collision terms to the solid nodes:

27 $$\Omega = \frac{1}{\tau }{(1-B)(f}^{eq}-f)+B{\Omega }^{S}$$

where *B* is determined by the solid ratio ε of a solid-occupied node:

28 $$B=\frac{\varepsilon (\tau -0.5)}{\left(1-\varepsilon \right)+(\tau -0.5)}$$

In addition, ${\Omega }^{S}$ describes the bounce-back of liquid hitting the particle:

29 $${\Omega }^{S}={f}_{-}\left({\boldsymbol{x}},t\right)-f\left({\boldsymbol{x}},t\right)+{f}^{eq}\left(\rho ,{{\boldsymbol{U}}}_{{\boldsymbol{s}}}\right)-{f}_{-}^{eq}(\rho ,{\boldsymbol{u}})$$

where ***U***_*S*_ is the solid node’s velocity at the next time step.${f}_{-}\left({\boldsymbol{x}},t\right)$ is derived by setting the ${e}_{i}$ to ${-e}_{i}$ in $f\left({\boldsymbol{x}},t\right)$. It describes the distribution function's bounced-back state when encountering a solid node.

Subsequently, the hydrodynamic force ***F***_*f*_ and torque ***T***_*f*_ experienced by a solid particle can be evaluated by:

30 $${{\boldsymbol{F}}}_{{\boldsymbol{f}}}=c h\left[\sum_{n}({B}_{n}\sum_{i}{\Omega}_{i}^{s}{e}_{i})\right]$$

31 $${{\boldsymbol{T}}}_{{\boldsymbol{f}}}=c h\left\{\sum_{n}\left[(x-{x}_{C})\times ({B}_{n}\sum_{i}{\Omega}_{i}^{s}{e}_{i})\right]\right\}$$

where *c* is the lattice speed, *h* is the unit lattice length, and *c* = *h/Δt,* with *Δt* being the time step in the LBM. In the equations, *n* is the number of lattices or nodes that are partially occupied by a solid particle, and *i* is a discrete direction of a lattice point. By summing up the impact of the liquid on each affected node, it is possible to get the overall hydrodynamic force applied to a particle.

LBM allows the macroscopic kinematic viscosity *u* and density *ρ* to be calculated from the particle distribution function. In our simulation, we further considered fluid turbulence in the model, as it becomes significant when the Reynolds number reaches 5,000 or larger [9]. In our simulation, the Reynolds number reached 4,939, near 5,000, and could increase further at higher paddle speeds. For this, a static Smagorinsky model is used to modify liquid viscosity based on liquid velocity.

Particle movement is handled by Newton’s law of motion, which is described below. Three significant forces are considered: ${{\boldsymbol{F}}}_{{\boldsymbol{c}}}$ is the contact force due to particle–particle collision. ${{\boldsymbol{F}}}_{{\boldsymbol{f}}}$ is the hydrodynamic force described in Eq. 30. The gravity is described as *mg*. ${T}_{c}$ and ${T}_{f}$ are the corresponding torques of ${F}_{c}$ and ${F}_{f}$. The particle mass and moment of inertia are *m* and *I,* respectively, while the acceleration and angular acceleration are *a* and $\ddot{\uptheta }$.

32 $$ma= {F}_{c}+{F}_{f}+mg$$

33 $$\mathrm{I}\ddot{\uptheta }= {T}_{c}+{T}_{f}$$

With the equations, particle translation and rotation can be calculated.

### DEM Modeling of Particle Dynamics

To model particle–particle interactions, we used DEM to simulate the behavior of bulk particles in the USP Apparatus II. There are various models for contact forces. Here in our model, Hertz-Mindlin contact force law is used [52].

34 $${\mathrm{F}}_{\mathrm{c}}={k}_{n}{\delta}_{{n}_{ij}}-{\gamma}_{n}{v}_{{n}_{ij}}$$

35 $${\mathrm{T}}_{\mathrm{c}}={k}_{t}{\delta}_{{t}_{ij}}-{\gamma}_{t}{v}_{{t}_{ij}}$$

where *k*_*n*_ and *k*_*t*_ are the elastic constants of normal and tangential contact. *δ*_*nij*_ and *δ*_*tij*_ are the normal and tangential overlap of the distance between two particles. *γ*_*n*_ and *γ*_*t*_ are the viscoelastic damping constants of normal and tangential contact. *v*_*nij*_ and *v*_*tij*_ are the relative velocities of the normal and tangential components.

The parameters of *k*_*n*_, *γ*_*n*_, *k*_*t*_, *γ*_*t*_ could be calculated as follows:

36 $${\mathrm{k}}_{\mathrm{n}}=\frac{4}{3}{Y}^{*} \sqrt{{R}^{*}{\delta}_{n}}$$

37 $${\upgamma}_{\mathrm{n}}=-2\sqrt{\frac{5}{6}} \beta \sqrt{{S}_{n}{m}^{*}} \ge 0$$

38 $${\mathrm{k}}_{\mathrm{t}}=8{G}^{*} \sqrt{{R}^{*}{\delta}_{n}}$$

39 $${\upgamma}_{\mathrm{t}}=-2\sqrt{\frac{5}{6}} \beta \sqrt{{S}_{t}{m}^{*}} \ge 0$$

The parameters of *S*_*n*_*, S*_*t,*_* and *β*,* can be calculated with the following equations:

40 $${\mathrm{S}}_{\mathrm{n}}=2{Y}^{*} \sqrt{{R}^{*}{\delta}_{n}}$$

41 $${\mathrm{S}}_{t}=8{G}^{*} \sqrt{{R}^{*}{\delta}_{n}}$$

42 $$\upbeta =\frac{\mathrm{l}\mathrm{n}(e)}{\sqrt{{\mathrm{ln}}^{2}\left(e\right)+{\pi }^{2}}}$$

43 $$\frac{1}{{\mathrm{Y}}^{*}} =\frac{1-{v}_{i}^{2}}{{Y}_{i}} + \frac{1-{v}_{j}^{2}}{{Y}_{j}}$$

44 $$\frac{1}{{\mathrm{G}}^{*}} =\frac{2(2+{v}_{i})(1-{v}_{i})}{{Y}_{i}} + \frac{2(2+{v}_{j})(1-{v}_{j})}{{Y}_{j}}$$

45 $$\frac{1}{{\mathrm{R}}^{*}}= \frac{1}{{\mathrm{R}}_{\mathrm{i}}}+ \frac{1}{{\mathrm{R}}_{j}}$$

46 $$\frac{1}{{m}^{*}}=\frac{1}{{m}_{i}}+ \frac{1}{{m}_{j}}$$

In the above equations, *e* is the coefficient of restitution, *Y* is Young’s modulus, *v* is the Poisson ratio, *μ*_*s*_ is the coefficient of static friction, *m* is the particle's mass, and *R* is the particle's radius. The subscripts of *i* and *j* stand for the two particles in contact. The superscript * of a value means the value calculated with particle *i* and *j* values.

### Simulation Configuration

**Fig. 3:**
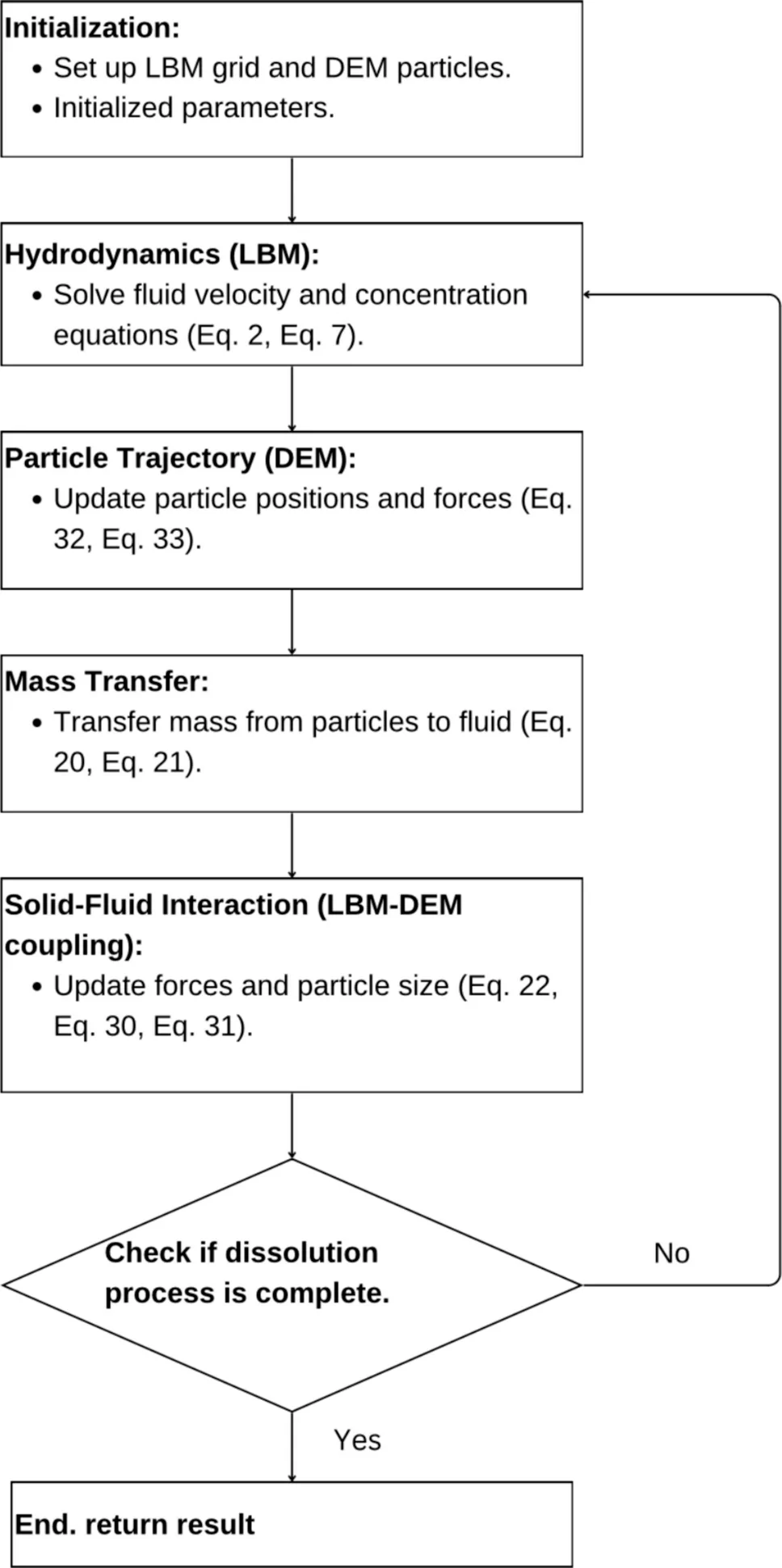
LBM-DEM coupling algorithm to simulate particle dynamics in liquid.

In our simulation that combines hydrodynamic and particle dynamics, the LBM component was implemented by using the open-source library Palabos (Parallel Lattice Boltzmann Solver) [53]. Particle dynamics were modeled with LIGGGHTS (LAMMPS Improved for General Granular and Granular Heat Transfer Simulations), an open-source DEM library [54]. Coupling was carried out using the open-source library LBDEMcoupling [55, 56]. The production runs were performed in a Linux command-line environment on the cluster described in 'Simulation Model Performance' below, utilizing C/C++-based solver libraries; the workflow did not rely on a specific IDE. The algorithm is shown in Fig 3.

The coupling library transfers particle parameters such as coordinates, velocity, and radius between the LBM and DEM solvers. Hydrodynamic force and torque on each particle are computed based on Eqs. 30 and 31 and sent back to the DEM solver as external loads that influence particle translation and rotation. The DEM parameters were taken from the literature and then fine-tuned to ensure numerical stability, as shown in Table I [57–59].

**Table I:** Parameters Used in LBM-DEM Simulation

Temperature (*T*)	37 °C
Fluid Density (*ρ*_*f*_)	993.3 kg/m^3^
Kinematic Viscosity (*v*_*k*_)	6.97e-7 mm^2^/s
Water Volume (*V*)	1 L
Particle radius (*R*)	500 μm
Particle density (*ρ*_*p*_)	1100 kg/m^3^
Number of particles (*N*)	~1,060
Young’s modulus (*Y*)	6.13e6 Pa
Poisson’s ratio (*ν*)	0.225
Coefficient of restitution (*e*)	0.8
Coefficient of static friction (*μ*_*s*_)	0.2
Timestep (dt)	6.67e-5(s)
Grid size	204 × 204 × 243
Paddle agitation speeds (RPM)	50, 60, 85, 100

### Model Validation and Grid Independence Study

To ensure the accuracy and reliability of our simulation model, we performed a comprehensive model validation and grid independence study.

#### Validation Against Experimental Data

**Fig. 4:**
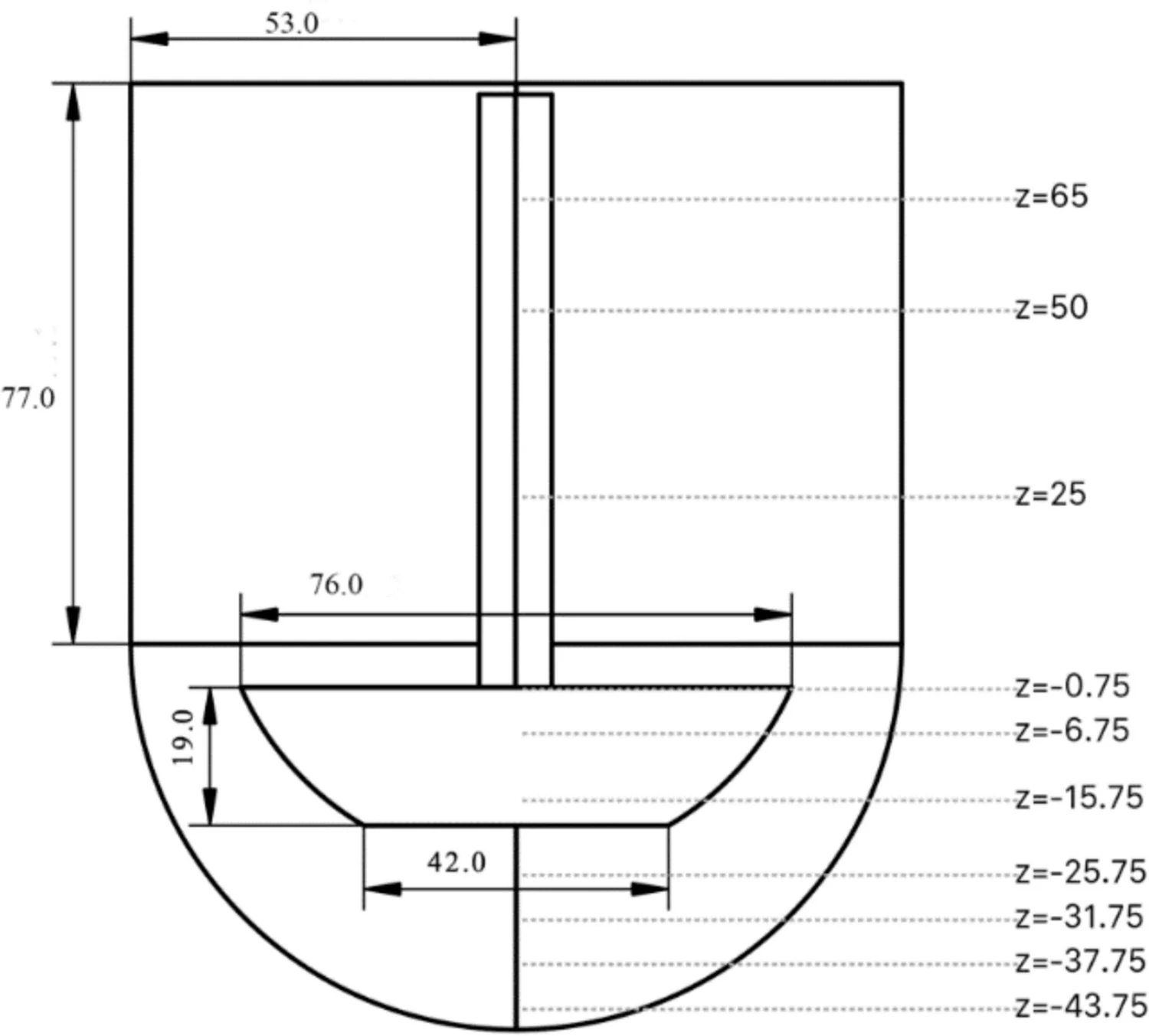
Configuration of USP Apparatus II with ten iso-surfaces along the z-axis selected for comparison with LDV data. All units are in mm.

**Fig. 5:**
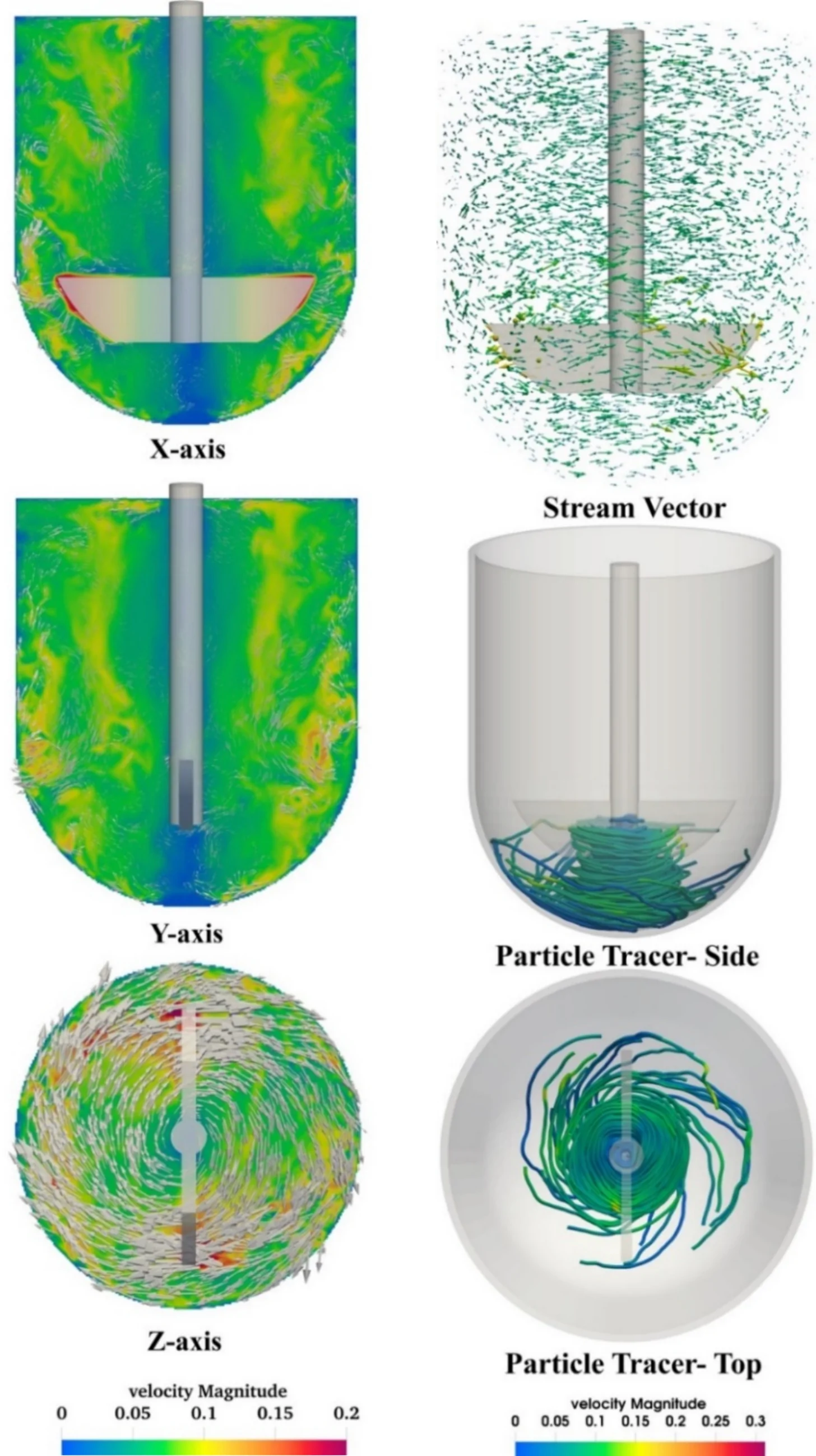
Left: Velocity distribution from X-axis, Y-axis, and Z-axis viewed under 50 rpm. Right top: Vector of stream velocity distribution in the whole USP Apparatus II. Right middle and bottom: particle movement tracer at the bottom of the USP Apparatus II. Velocity magnitude is in units of m/s.

We validated our LBM model by comparing the simulated fluid velocity profiles with experimental measurements [10], which utilized Laser Doppler Velocimetry (LDV) to measure fluid velocities in the USP Apparatus II. The experimental setup included a paddle rotation speed of 50 rpm and measured velocities at ten horizontal planes within the vessel (Figs. 3, 4 and 5).

**Fig. 6:**
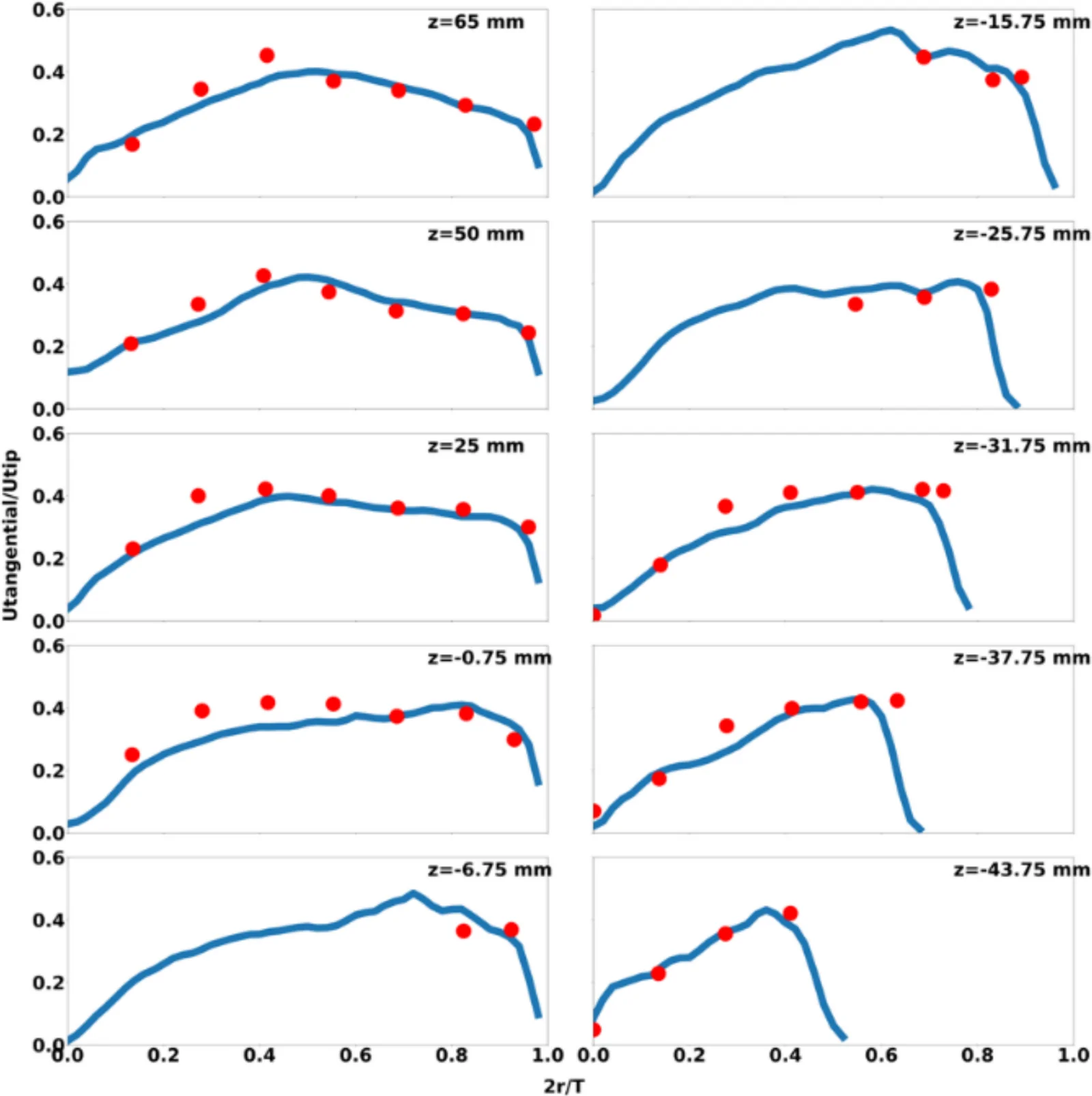
Comparison between LDV data and LBM predictions of tangential velocities on different iso-surfaces. Blue line-simulation results, red dots -LDV data (from reference [10]).

**Fig. 7:**
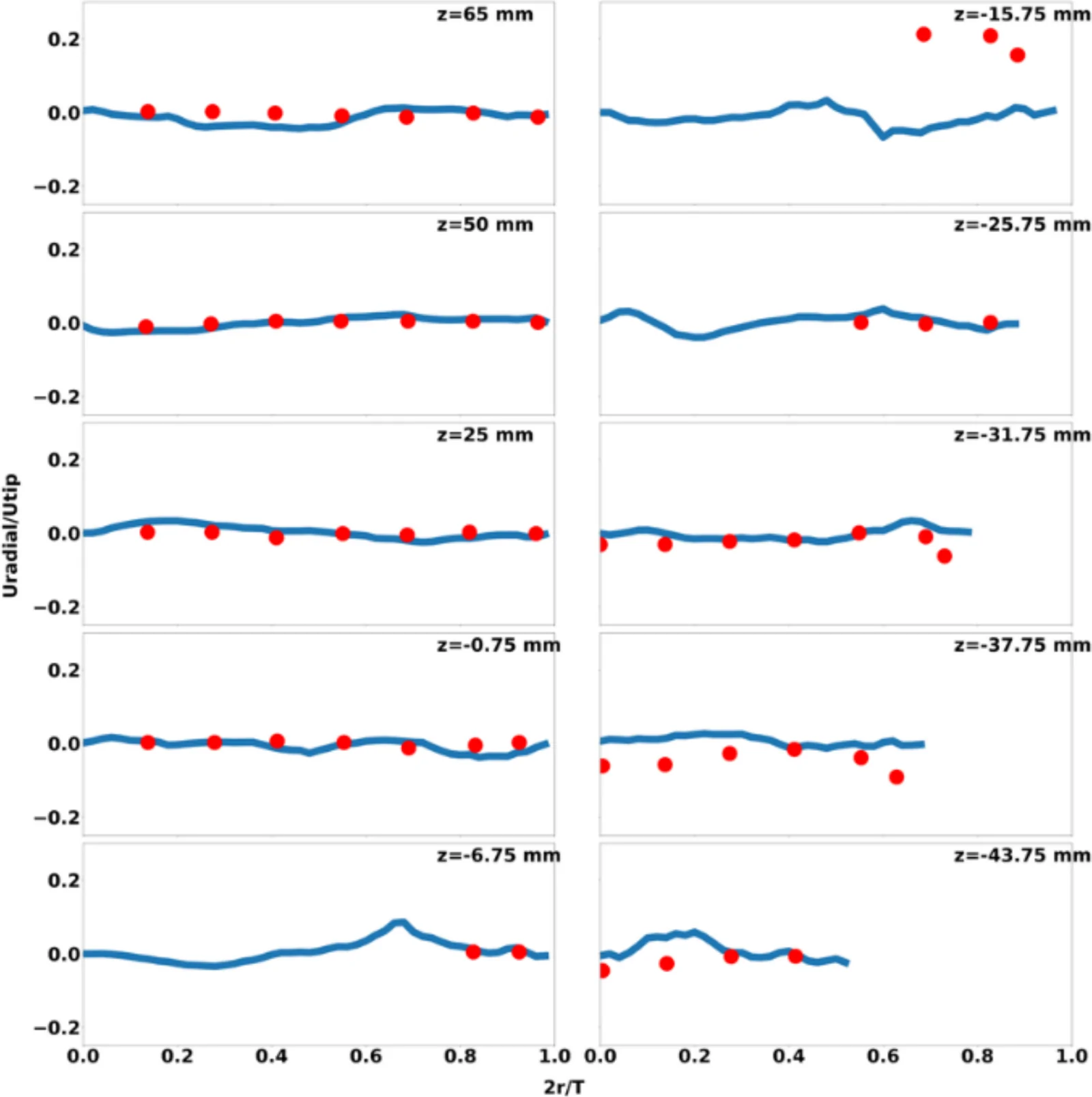
Comparison between LDV data and LBM predictions of radial velocities on different iso-surfaces. Positive values indicate fluid moving out of the vessel center for radial velocity. Blue line—simulation results, red dots—LDV data (from reference [10]).

**Fig. 8:**
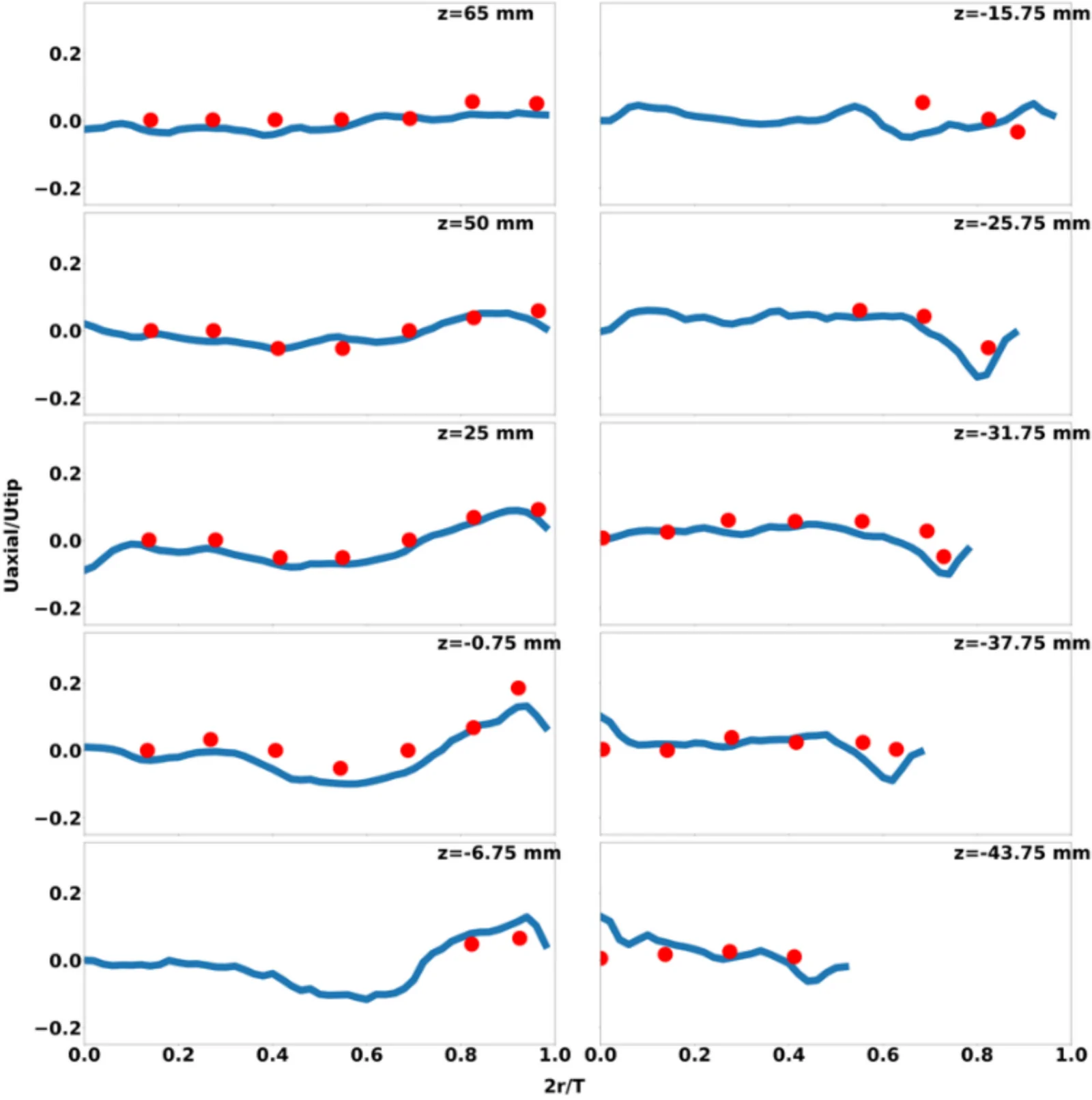
Comparison between LDV data and LBM predictions of axial velocities on different iso-surfaces. For axial velocity, positive values indicate fluid moving up. Blue line—simulation results, red dots—LDV data (from reference [10]).

We replicated these conditions in our simulation and extracted the velocity profiles at corresponding positions. Figures 6, 7 and 8 compare our simulation results with the experimental data for tangential, radial, and axial velocities, respectively. The close agreement demonstrates that our LBM model accurately captures the hydrodynamic behavior within the apparatus, thereby validating it against experimental results.

For the single-particle dissolution validation, the overall agreement with the literature data is qualitative rather than exact. The remaining differences are due to several simplifying assumptions in the current model, including idealized spherical particles, a constant intrinsic dissolution rate, a constant molecular diffusion coefficient, and the neglect of surface roughness, particle polydispersity, and any transient agglomeration or breakup. We now explicitly state these limitations so that the validation is understood at the appropriate level.

#### Grid Independence Study

To ensure that our simulation results are not dependent on the grid resolution, we conducted a grid independence study using three different grid sizes:

- Coarse Grid: 102 × 102 × 122
- Medium Grid: 204 × 204 × 243 (used in the main simulations)
- Fine Grid: 306 × 306 × 363

We evaluated key parameters such as fluid velocity profiles, shear rates, and particle dissolution rates across these grids. The timestep was adjusted for each grid to satisfy the CFL condition. Our findings indicated that the differences between the medium and fine grids were negligible, with less than 1% variation in velocity and particle dynamics. This confirms that the medium grid provides sufficient resolution for accurate simulations while maintaining computational efficiency.

#### Timestep Selection

The timestep (**Δt** = 6.67 × 10^−5^ s) was selected based on stability and accuracy considerations. We conducted sensitivity analyses by testing smaller and larger timesteps to ensure numerical stability and accurately capture the physical processes (fluid flow and particle dynamics). The chosen timestep satisfies the stability criteria for both LBM and DEM in the simulation.

## Results and Discussion

Simulations were conducted based on the coupling approach described in the previous section. The first task was to perform only LBM calculations of hydrodynamics in the USP Apparatus II model and compare the computed fluid velocities with experimental values reported in the literature [10]. The second task was to run coupled LBM-DEM simulations and examine how the hydrodynamics determined particle dynamics in the apparatus.

### LBM Simulation of Fluid in Dissolution Device

Using the simulation parameters shown in Table I, we conducted LBM simulations without any solid particles present. Figure 5 shows the calculated results of liquid velocities in USP Apparatus II under 50 rpm after 300 s from the initial static state. The color intensities at the bottom of the panel correspond to the velocity scale. In contrast, the length and color of the streamlines in the right panel are commensurate with the velocity scale and direction. Liquid flow mainly occurs away from the vessel's vertical center, where liquid velocities are lowest. The paddle tip has the highest speed, driving fluid movement. To track the fluid dynamics, we placed pseudo particle tracers at the bottom of the apparatus, and we observed that the fluid would move alongside the internal boundary of the vessel and move upward to the paddle, forming a low shear region [17].

We further compared velocity values with those determined experimentally by LDV in a previous study by Bai et al. [10]. As shown in Fig. 4, we extracted velocity values at ten different positions along the z-axis from the simulation result and plotted them along with the LDV data. We decomposed each velocity component into tangential, radial, and axial components and compared the resulting scales with experimental values. Figure 6 shows tangential velocities from the simulation, superimposed with the literature data. We generally observe an increase in tangential velocity from the center to the vessel wall along each horizontal line. Note that the origin along the X-axis of each figure is at the vessel center—the increase in tangential velocity results from the increasing velocity of the paddle along its axis. Along the vertical axis of the vessel from top to bottom, the most prominent position of velocity seems to shift from the center of the figure (or a quarter from the wall) towards the wall. Velocity values closely resemble those observed experimentally, especially the trends along the horizontal and vertical directions.

Figures 7 and 8 show radial and axial velocity values calculated from the LBM simulation. The radial velocities are near zero; most values match experimental data, except for the z position at −15.75 mm. Given that other data points are closely matched, we suspect the deviation may be due to experimental errors arising from possible interference between paddle movement and the laser measurement. For the axial velocity distribution (Fig. 8), most simulated values fluctuate around zero, especially at horizontal positions further away from the paddle (z = 65 and 50 mm). Upward flow can be seen near the wall at places above the paddle (z = 25, −0.75, and −6.75 mm). The upward flow is also observed at the bottom of the apparatus (z = −43.75 mm). The half-moon shape of the paddle induced different flow patterns (Fig. 4). The velocity on the paddle’s top tip is higher than at its bottom tip. Based on Bernoulli’s principle, the fluid will move upward from the lower part of the vessel to the upper part. This force, we posit, could contribute to the dissolution of the tablet in the dissolution vessel by facilitating particle dissolution on the top of the tablet.

### Particle Dynamics under Hydrodynamics

**Fig. 9:**
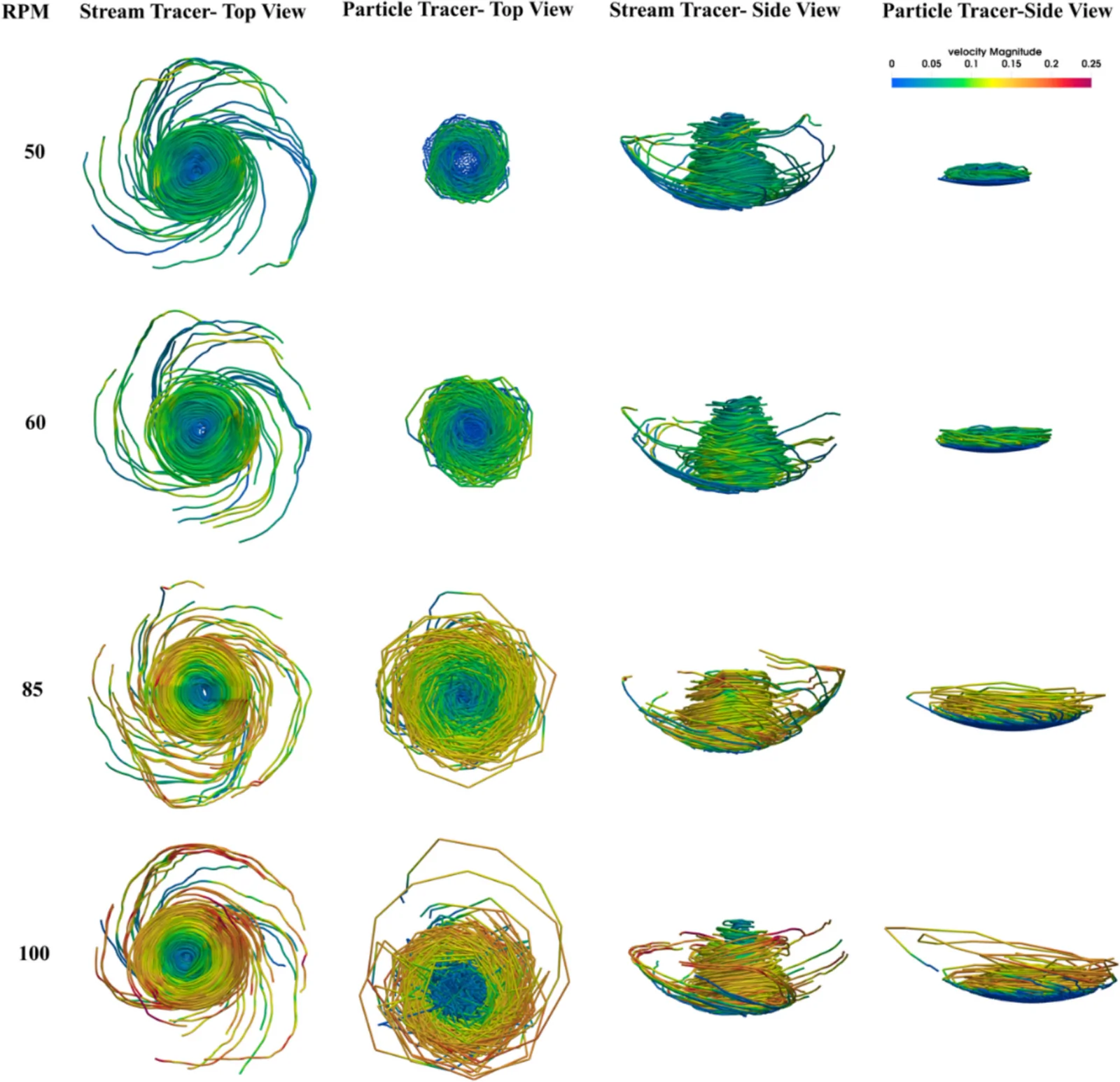
Comparison of fluid and particle movement at different RPMs. The stream tracer shows the fluid motion, as indicated by pseudo-particles placed at the bottom of the vessel. The particle tracer shows particle movement by tracking the drug particles. Velocity magnitude is in units of m/s.

To balance simulation accuracy and computational demand, about 1,000 solid particles were simulated in the USP Apparatus II with 1 L of liquid at various paddle rotational speeds. Table I lists the computational parameters for the coupled DEM-LBM simulation. Figure 9 shows both the fluid dynamics and particle velocities after 250 s from the initial state, in which particles were kept static at the center of the vessel bottom. We traced particle movement for 100 s, allowing several observations.

**Fig. 10:**
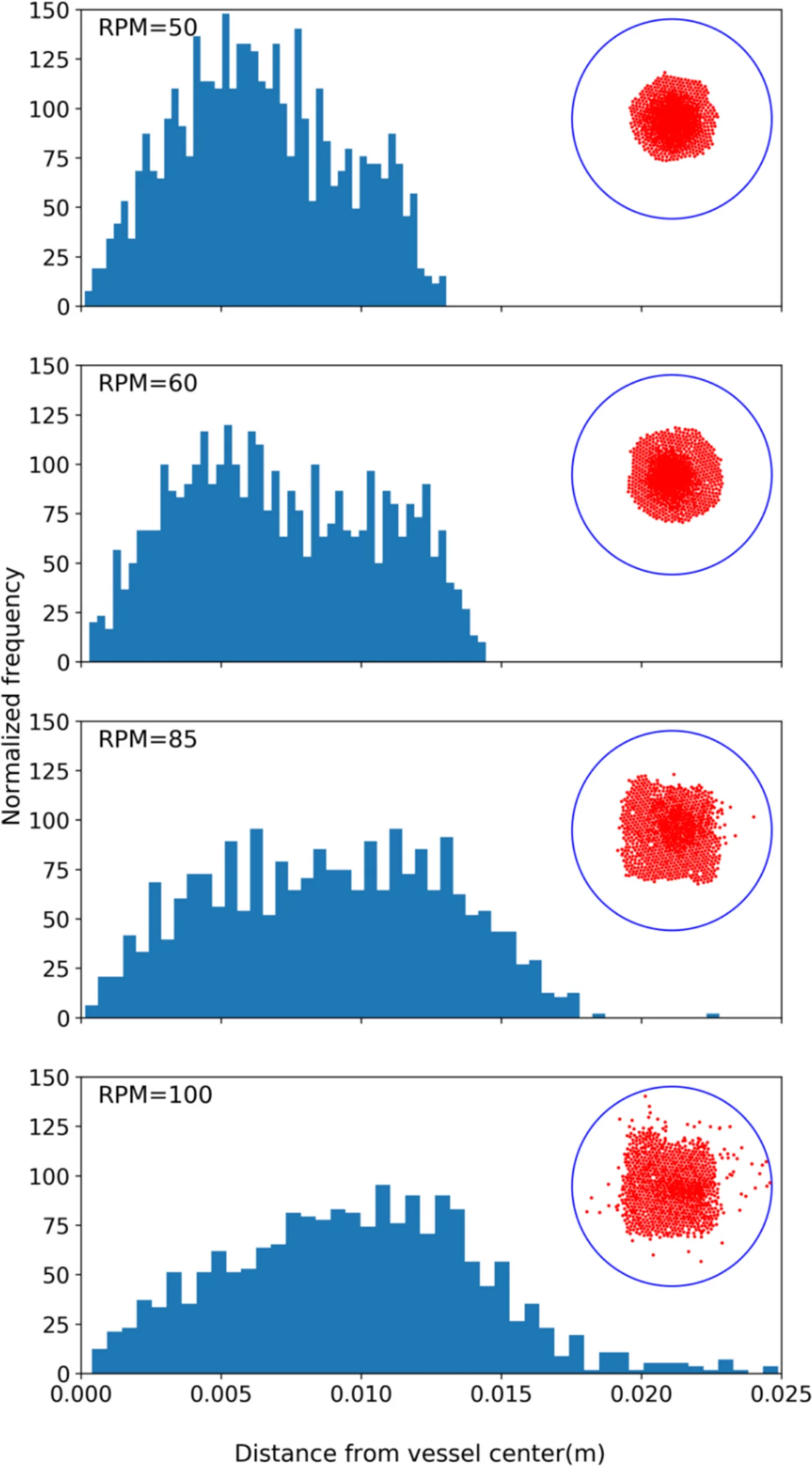
Comparison of particle distribution histogram at different rotating speeds.

First, the streamlines of fluid show that liquid velocity positively correlates with the paddle’s rotational speed. The fluid tracing also suggests upward movement from the bottom of the vessel. Particle velocities are significantly influenced by the paddle’s stirring rate. At 50 or 60 rpm, all particles are concentrated at the bottom of the vessel. Particle path lines converge toward the center. As the paddling speed increases, particle velocities and spatial distributions become more evident, as illustrated by the streamlines (Fig. 9). Particle path lines diverge, indicating particles move away from the vessel center. Thicker path lines show that particles also move faster at higher paddle speeds. This movement creates the so-called “coning effect,” where particle aggregation occurs at the bottom of the dissolution vessel due to insufficient agitation. From the fluid dynamics illustrations (Figs. 5 and 9), it is clear that fluid velocities along the center axis are much smaller, which contributes to solid particle settling. The coning effect diminishes as paddle speed increases, as shown in Fig. 10. The histogram of particle frequency, or the number of particles within a unit area along the horizontal axis from the vessel's center, shows that the particle distribution becomes flatter and extends toward the wall at higher paddling speeds. The inset images in Fig. 10 also confirm this trend of broader spatial distributions as the stirring rate increases from 50 to 100 rpm. This aligns with the empirical expectation that increasing paddle speed disrupts cone formation and encourages particle resuspension.

The cone structures observed here should also be interpreted carefully. In dissolution practice, visually similar cone shapes may represent either 'bad' cones, which trap material and hinder release, or 'good' cones, which maintain a cone-like shape while still allowing effective release. Since the current simulations do not yet incorporate excipients, pore development, or matrix trapping, they capture hydrodynamic cone formation but cannot yet differentiate all mechanistic pathways that distinguish benign from release-limiting cones.

For the same reason, we did not report a coning entrapment fraction as a definitive formulation metric in the current manuscript. Such a metric would be valuable, but in an API (Active Pharmaceutical Ingredient)-only compact, it would mainly reflect hydrodynamic retention rather than excipient-mediated entrapment. Therefore, we present cone-related results as hydrodynamic indicators and consider quantitative entrapment analysis across paddle speeds an important extension for future work.

There is literature to empirically derive the minimal rotation speed from fluid properties by which the coning phenomena disappear, NC_rpm_ (no coning rpm), by the Zwietering equation [60, 61].

47 $${\mathrm{N}\mathrm{C}}_{rpm}=A{d}_{p}^{l}{\left(\frac{{\rho}_{p}-{\rho}_{f}}{{\rho}_{f}}\right)}^{m}{\left(\frac{{\mu}_{f}}{{\rho}_{f}}\right)}^{n}$$

**Fig. 11:**
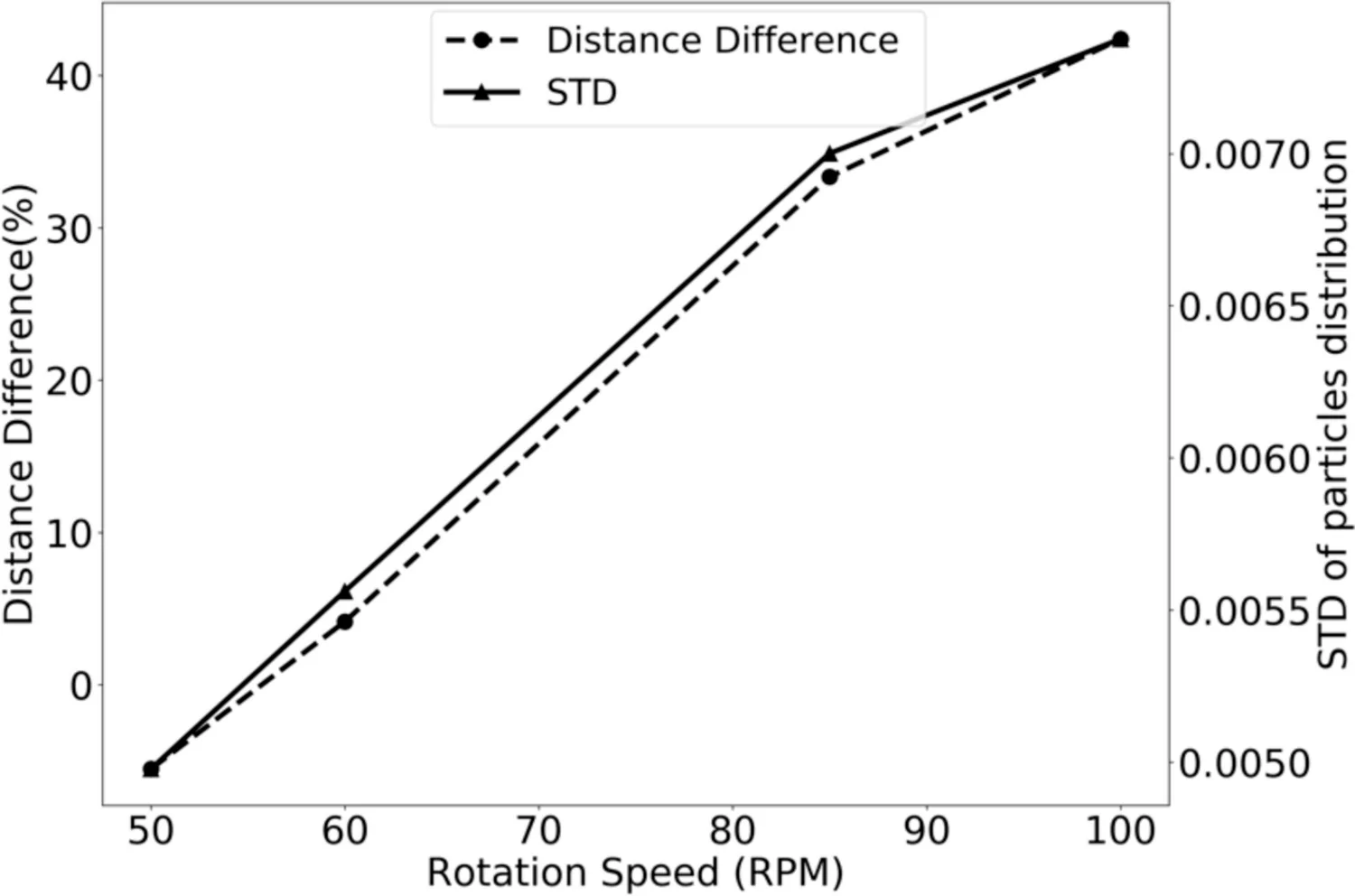
Relationship between standard deviation (STD) of particle distribution and USP Apparatus II paddle rotation speed. The change in the distance is evaluated between the initial state and the state 250 s after the simulation starts.

Studies report that for non-viscous fluids, such as dilute aqueous solutions (< = 2.1 mPa·s), the constant parameters A, l, m, and n are found to be 57, 0.22, 0.52, and −0.23, respectively [60–62]. For our simulations in which the diameter of the particle d_p_ = 1,000 μm, particle density ρ_p_ = 1.1 g/cm^3^, fluid density ρ_f_ = 1.0 g/cm^3^, and fluid viscosity μ_f_ = 0.692 mPa·s, NC_rpm_ is calculated as 85 rpm. From Fig. 10, particles disperse more widely at 85 rpm. The coning effect could be more drastic if we include more particles in the simulation. For a further quantitative assessment of particle motion, the standard deviations (STD) of particle coordinates and the changes in distance from the initial state to the vessel center were calculated and plotted together as a function of paddling speed (Fig. 11). Interestingly, the rotation speed determines both quantities linearly. Because of the size and geometry limitations of the USP Apparatus, the distance difference and STD are capped. These two values will not increase linearly alongside the RPM.

The simulation results indicate that particles tend to settle to the bottom of the dissolution vessel, resulting in higher local concentrations. This non-uniform distribution of dissolved drugs can drastically affect sampling results, particularly when samples are taken midway through the vessel, as is practiced in standard dissolution testing protocols. Our results suggest that the midway sampling underestimates the actual concentration of the dissolved drug, especially at early times when dissolution occurs rapidly at the bottom. This finding could shed light on dissolution testing, highlighting the need to consider different sampling positions.

### Dissolution of Bulk Particles in USP Apparatus II

Several studies have examined the effect of paddle speed and other hydrodynamic conditions on particle and tablet dissolution in the Apparatus [6, 9, 63]. Similarly, we performed calculations to simulate various scenarios, including changes in paddle rotation speed and the particle dissolution kinetic constant K, to evaluate our model’s feasibility and accuracy. In this study, the diffusion coefficient for sodium carbonate was taken from literature-consistent aqueous values at 37°C, and the dissolution constant was chosen to match the expected single-particle release timescale before applying it in the tablet-level simulations.

These inputs should therefore be considered material-specific but still simplified parameters. A more rigorous predictive workflow would determine D independently from physicochemical data and calibrate K using dedicated intrinsic or single-particle dissolution experiments for the exact material system studied.

**Fig. 12:**
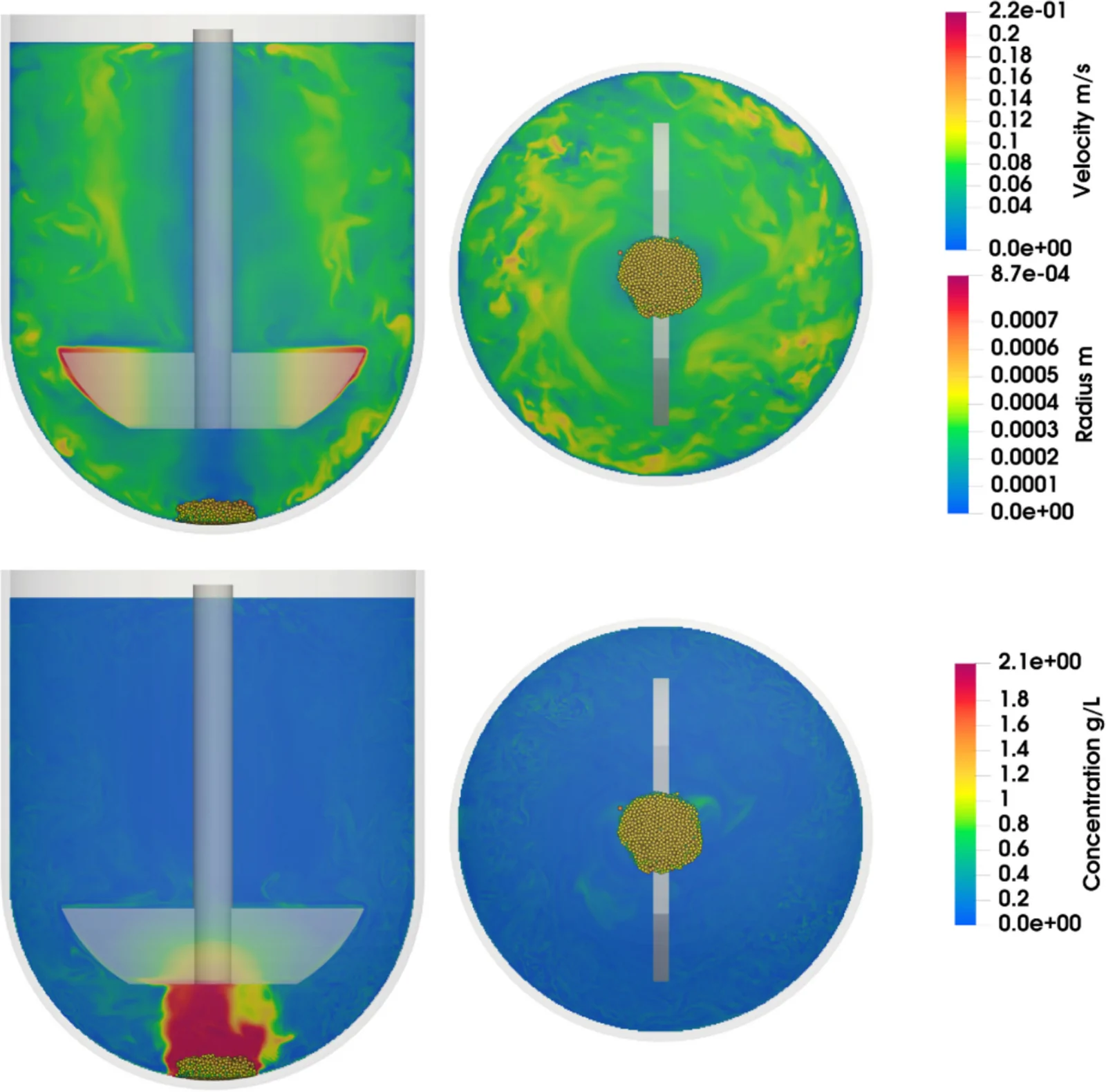
Velocity field (top row) and concentration field (bottom row) of dissolution simulation at 20 s, viewed from side and top. Particles compressed in a tablet are placed at the bottom of the USP Apparatus II.

**Fig. 13:**
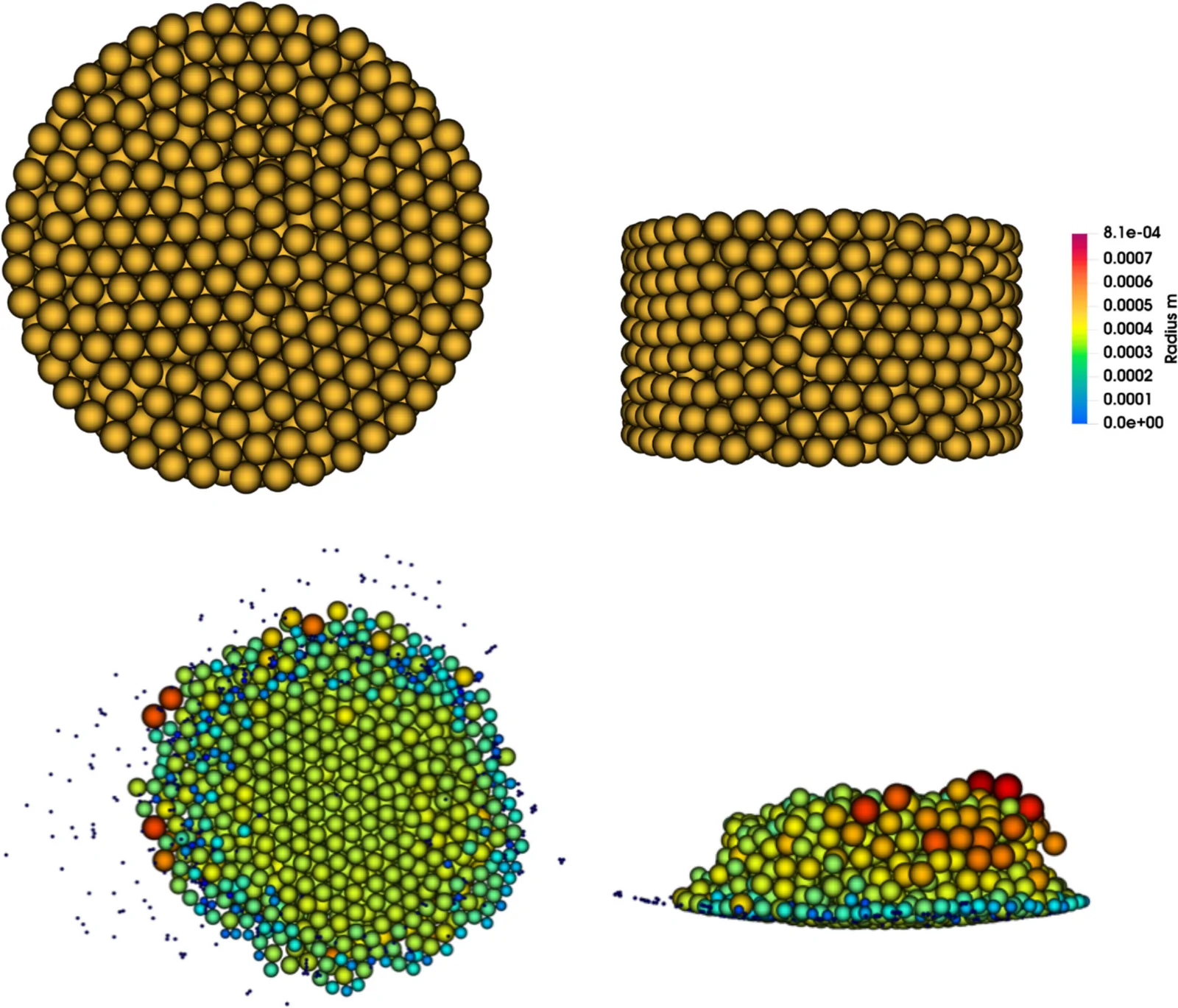
Dissolving particles at the beginning of the simulation (top row) and at 20 s (bottom row), viewed from the side and top. The color is indicative of a particle’s radius.

As shown in Fig. 12, USP Apparatus II is modeled using the lattice Boltzmann method, and the dimensions stem from USP general chapter 711 [1]. To validate our model, we used sodium carbonate as the drug particle. About 2,000 particles with a radius of 500 μm are compressed into a tablet via DEM simulation (Fig. 13). These particles are first loaded into a punch mold, and then a punch die is used to press them into a cylindrical tablet. The average overlap between particles is 10% of the radius. This DEM-modeled tablet is placed at the bottom of the USP Apparatus II. The simulation parameters are listed in Table II. Figure 12 shows the velocity and concentration fields, respectively, indicating a highly concentrated area under the paddle at the bottom of the Apparatus. Figure 13 shows the dissolving particles, suggesting that those at the surface dissolve faster than those in the tablet's core. The particles on the side also dissolve faster than those at the top.

**Table II:** Parameters of sodium carbonate used for USP Apparatus II simulation

Temperature (*T*)	37 °C
Fluid Density (*ρ*_*f*_)	993.3 kg/m^3^
Water Kinematic Viscosity(*v*_*k*_)	6.97e-7 m^2^/s
Diffusion Coefficient (D)	1.12e-9 m^2^/s
Dissolution constant (K_0_)	1.0e-5 m/s
Water Volume (*V*)	850 mL
Particle radius (*R*)	500 μm, 1500 μm
Particle density (*ρ*_*p*_)	2540 kg/m^3^
Number of particles (*N*)	100 ~ 2000
Young’s modulus (*Y*)	6.13e6 Pa
Poisson’s ratio (*ν*)	0.225
Coefficient of restitution (*e*)	0.8
Coefficient of static friction (*μ*_*s*_)	0.2
Loading stiffness (*k*_*1*_)	1.e5
Unloading stiffness (*k*_*2*_)	4.e6
Adhesion stiffness (*k*_*adh*_)	5.e4
Adhesion Exponent (*n for k*_*adh*_*)*	10
Overlap Exponent (*n for k*_*1*_* and k*_*2*_*)*	1.5
Timestep (dt)	6.67e-5(s)
Grid size of the model	306 × 306 × 363
Paddle agitation speeds (RPM)	40, 50, 85

The current framework can, in principle, represent different tablet solid fractions and drug loadings. This is because the initial DEM-generated compact can be modified by varying factors such as particle count, particle size distribution, die filling, compaction endpoint, and the ratio of dissolving to non-dissolving particles. However, this manuscript does not include a systematic study of those variables, so we refrain from making formulation-specific claims beyond the cases simulated here.

#### Dissolution Process

**Fig. 14:**
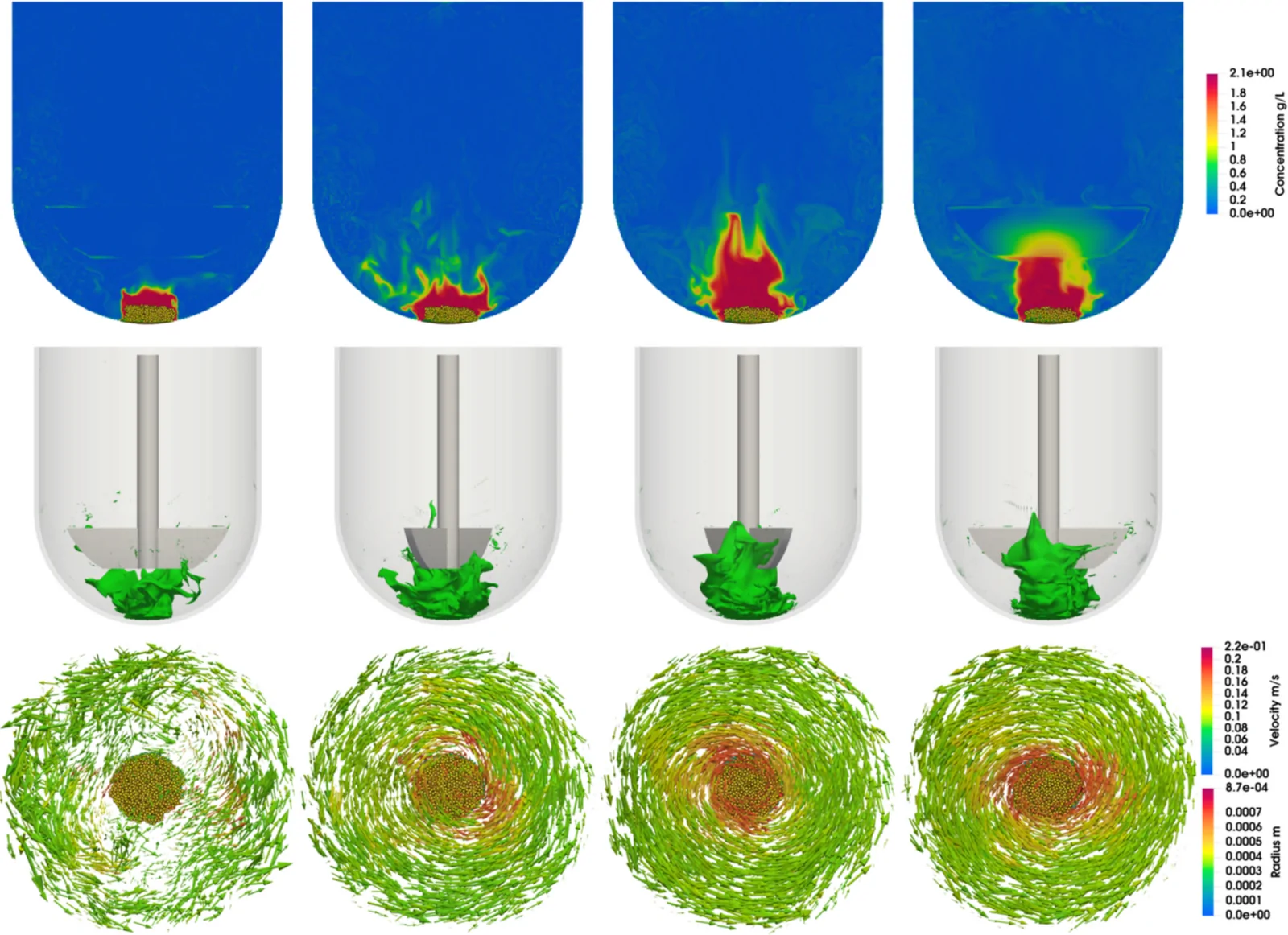
Dissolution process simulated at 5 s (first column), 10 s (second), 15 s (third), and 20 s (fourth). The first row shows the drug concentration distribution; the second row shows the contour of 1 mg/mL concentration; and the third row shows vector plots of the velocity field.

**Fig. 15:**
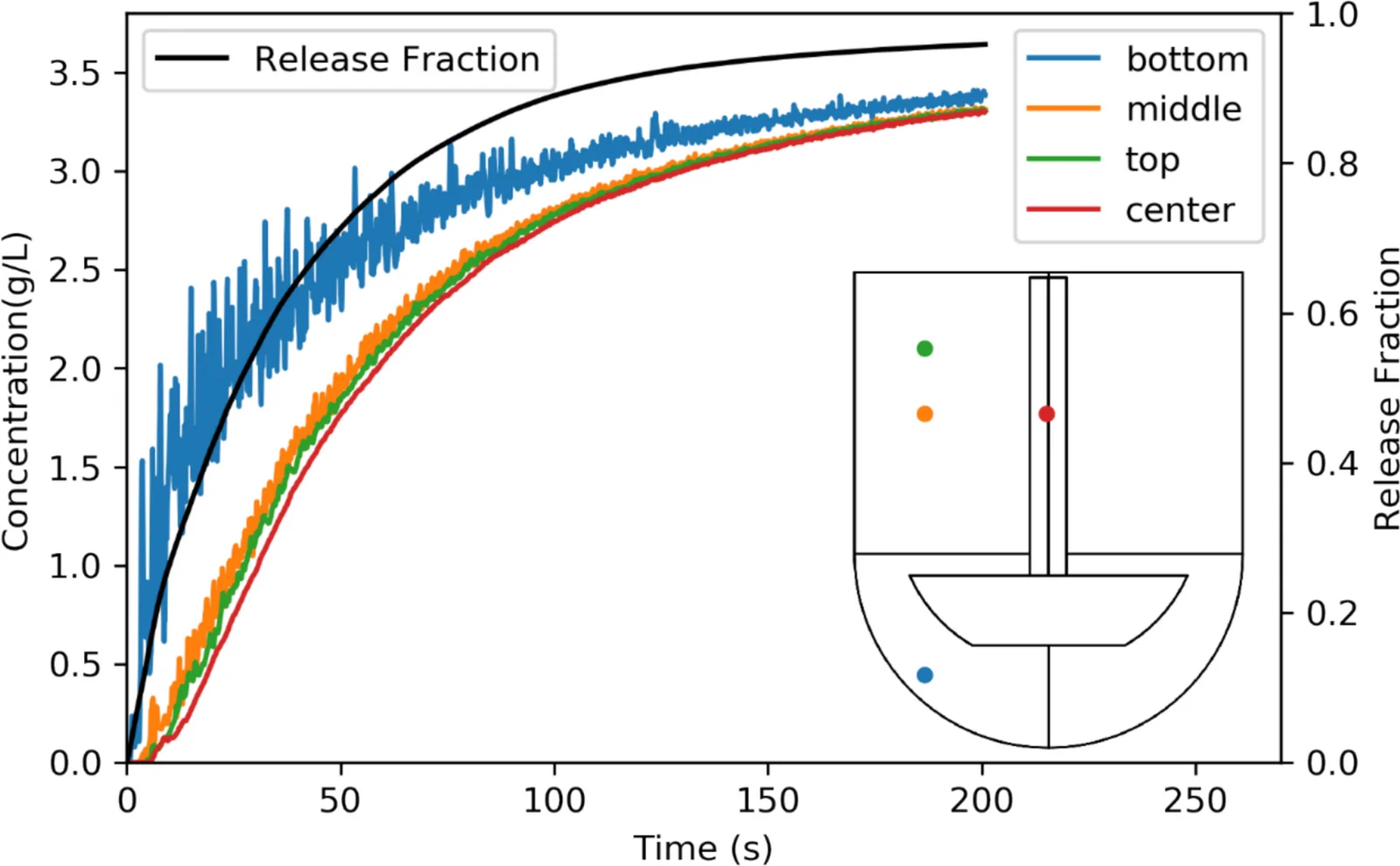
Concentration over time was sampled at four positions in the USP Apparatus II. The sampling positions are shown in the inset with the same colors as the plotted lines.

Visualizing particle dissolution enables us to closely examine the distribution of dissolved drugs in the liquid and how it is influenced by stirring in the USP Apparatus II. Figures 12 and 13 show that the dissolved drugs are concentrated immediately under the paddle and trickle up. The dissolution process is further revealed in Fig. 14, where the concentration field evolves. At first, dissolved drugs are localized under the paddle. As the paddle moves, the drug concentration begins to distribute toward the outer side of the vessel, leaving the inner region above the blades, to some extent, depleted of dissolved drugs (top row; Fig. 14). The observation hints that sampling in USP Apparatus II is highly heterogeneous. The concentration distribution over time is further examined in Fig. 15 at four different sampling locations. The concentration at the bottom spot increases fast and varies significantly due to the dynamic hydrodynamics in the region (bottom row; Fig. 14). For the two sampling spots in the middle, the concentration closer to the vessel wall (orange spot) is higher than that near the paddle rod (red), which shows the least variation in concentration. Interestingly, the concentrations at the three sampling spots above the paddle blades converge to the same value after 200 s. Note that the orange spot corresponds to the USP Chapter 711 sampling recommendation [1].

#### Vessel Hydrodynamics

**Fig. 16:**
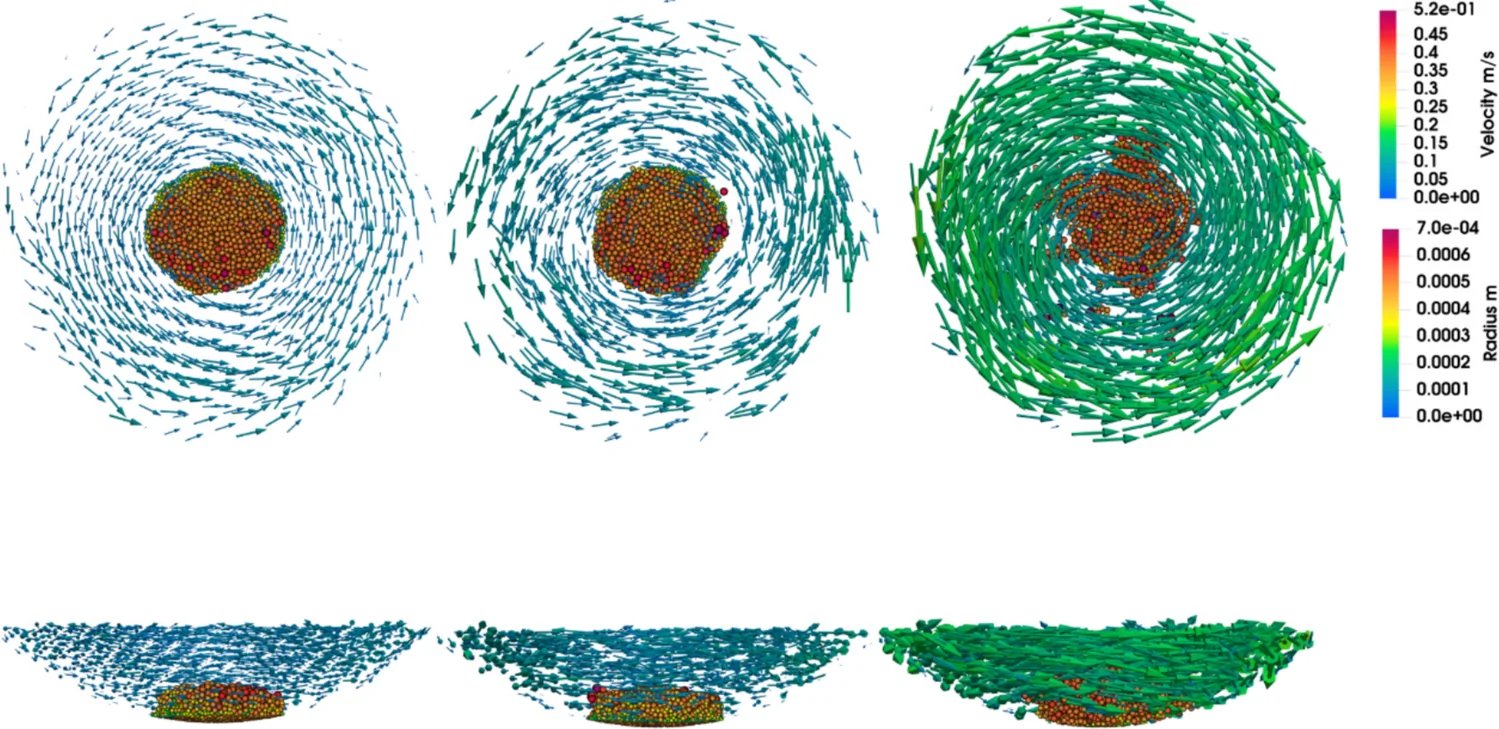
Velocity fields at three different stirring speeds: 40 RPM (left column), 50 (middle), and 85 (right), viewed from the top (top row) and the side (bottom). The size and color of a vector indicate the magnitude of the velocity at that location.

Fluid velocity fields near the particles in the Apparatus under three different paddle rotating speeds are shown as vectors in Figure 16. Tangential flow appears to form near the particles. As higher velocity induces stronger convection, this observation explains faster dissolution of particles on the tablet's side. Moreover, as the rotation speed increases, the velocity near the tablet increases, thereby promoting dissolution. At RPM = 85, the particles are distributed more widely than at lower RPMs. A higher stirring rate facilitates dissolution due to increased convection and reduced coning. Particles have more surface area for contacting the fluid when driven wider at the bottom of the apparatus. Collectively, a higher dissolution rate is observed at a faster stirring speed.

**Fig. 17:**
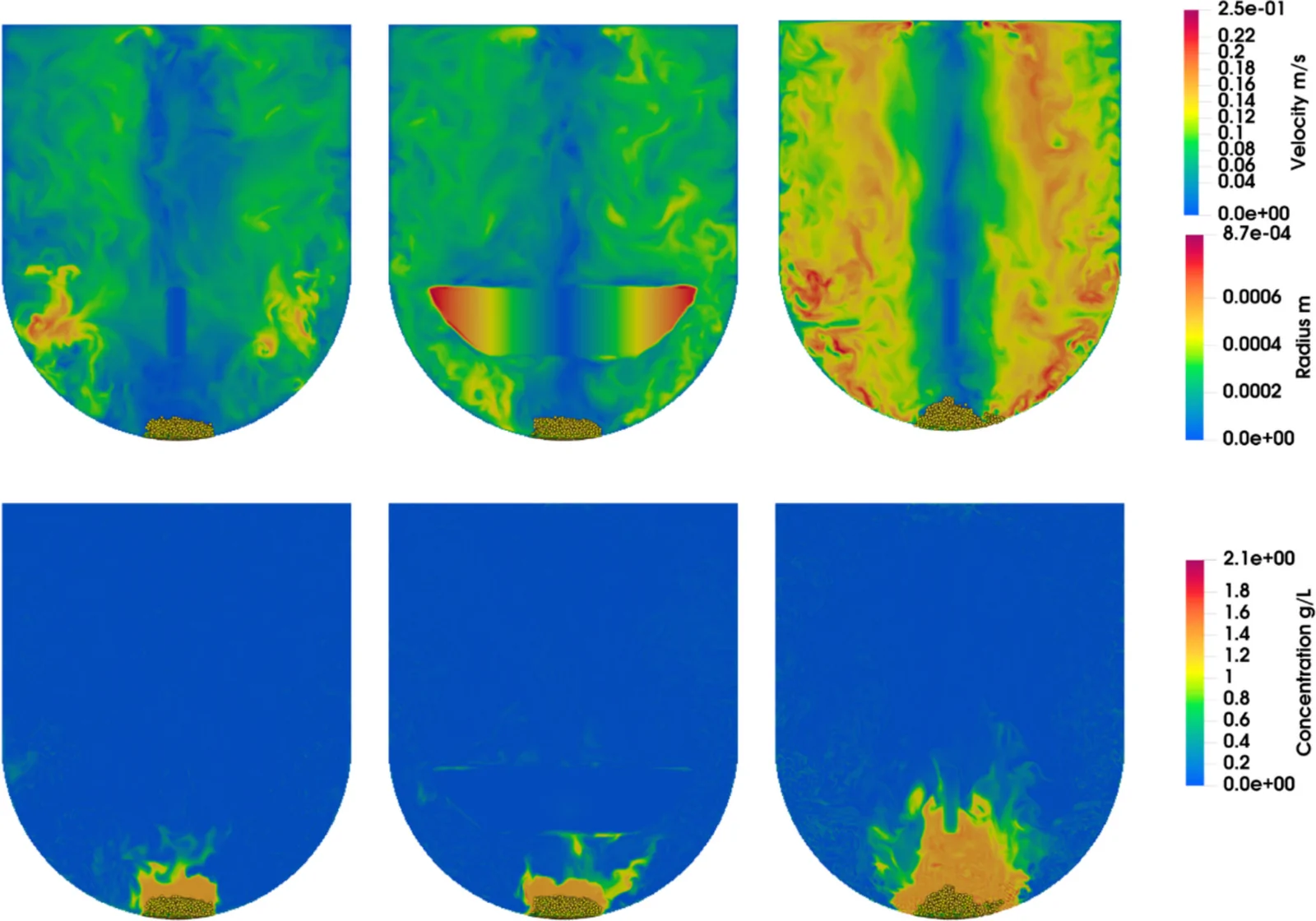
Concentration (top row) and velocity distributions (bottom) in USP Apparatus II simulated at time = 8 s under 40 RPM (left column), 50 (middle), and 85 (right).

**Fig. 18:**
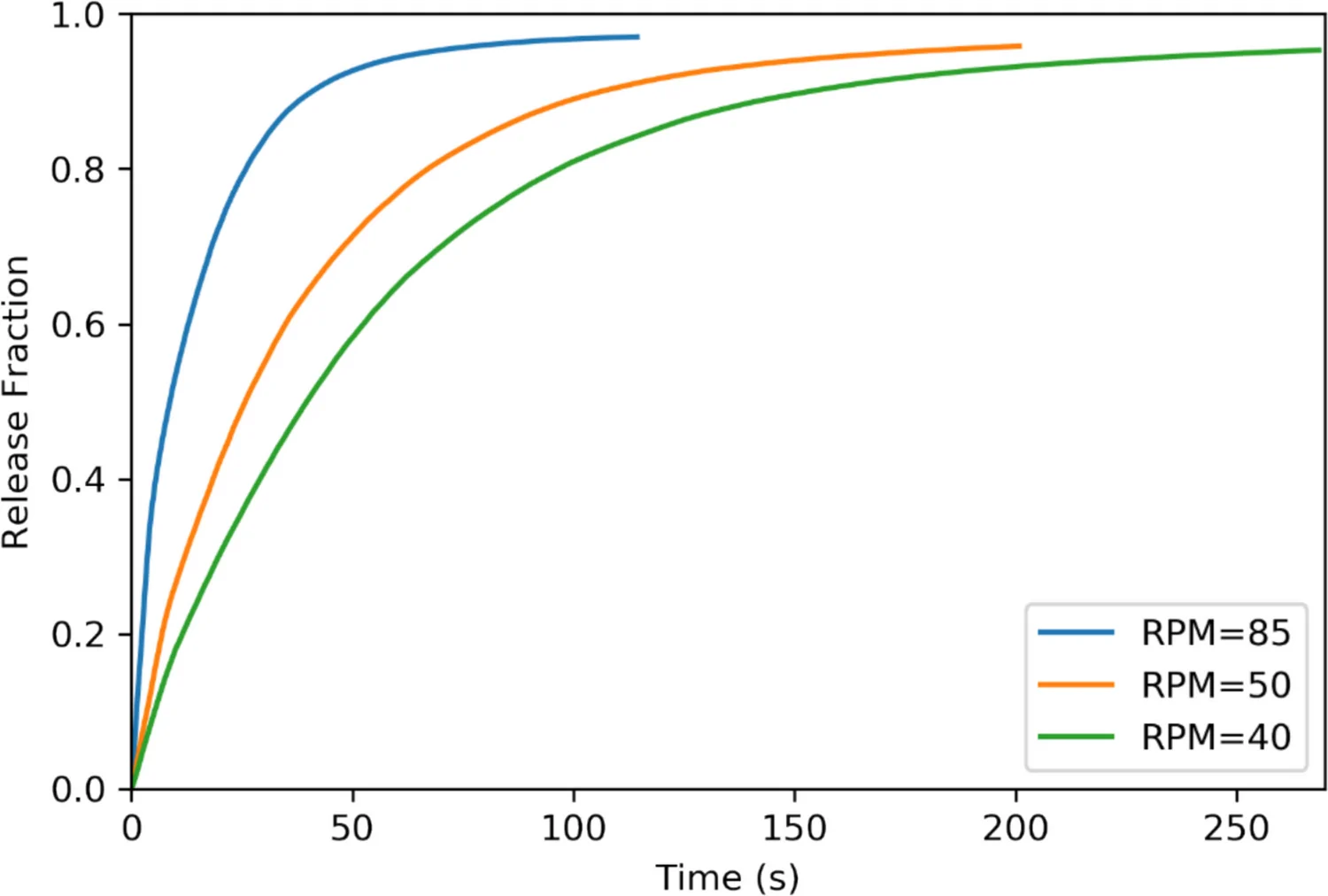
Concentration profiles under different rotation speeds.

The impact of stirring speed on tablet dissolution is illustrated in Fig. 17. A higher rotation speed induces higher velocity and faster particle dissolution. The velocities near the vessel wall are higher than those in the middle of the vessel. The noticeable low-velocity region in the middle is likely caused by the Apparatus configuration. As the stirring speed increases, the overall velocity increases, facilitating the dissolution of drug particles. Also noticeable is the high-concentration region at the bottom of the Apparatus spreading wider under a higher stirring rate because of the elevated convection. Additionally, the concentration profiles at 40, 50, and 85 RPM are shown in Fig. 18, further indicating the significant factor of paddle speed on particle and tablet dissolution.

#### Impact of Particle Dissolution Rate

**Fig. 19:**
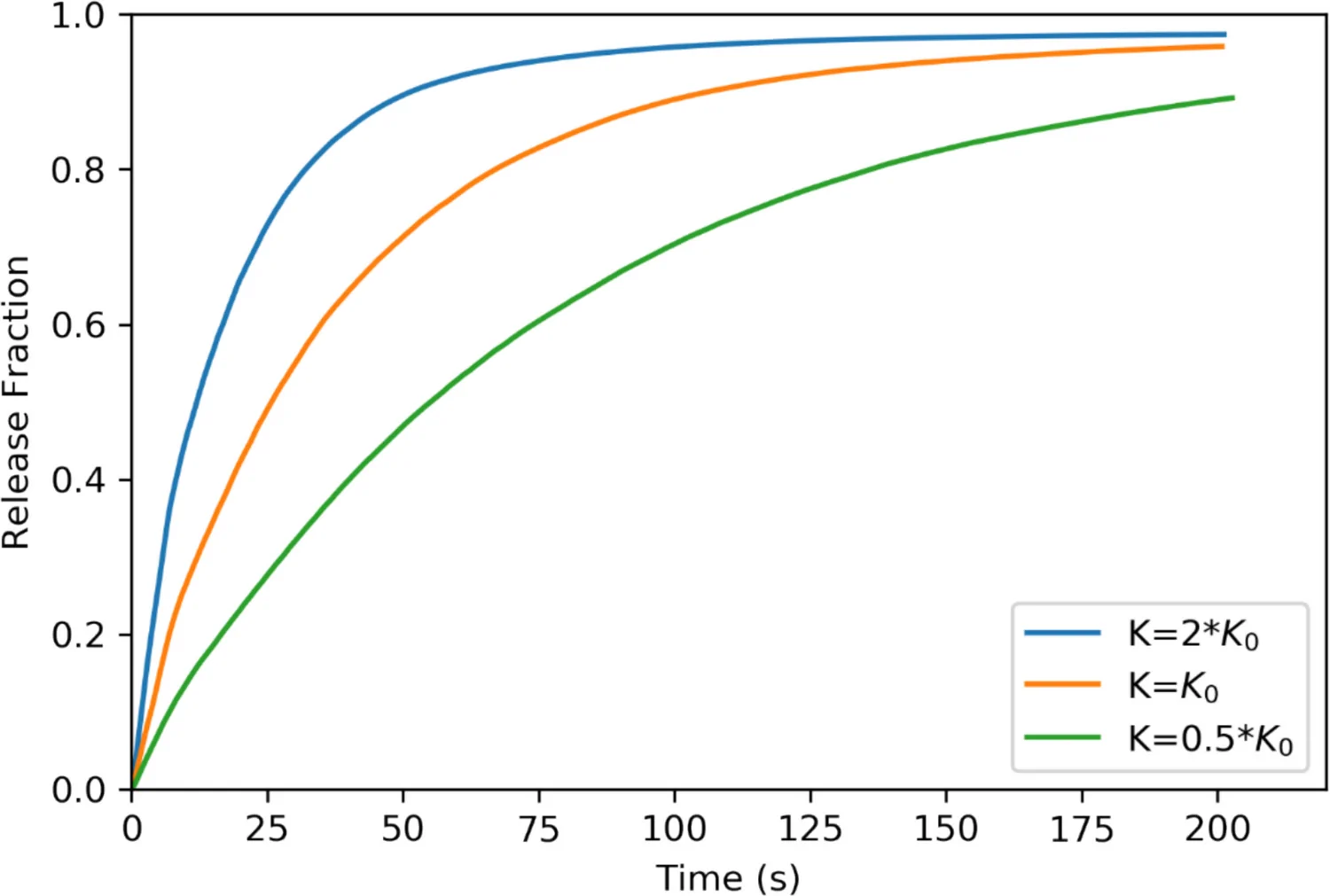
Concentration profiles of drug particles with different dissolution constant *K; K*_*0*_ is the constant of sodium carbonate (Table II).

To examine how drug solubility affects dissolution kinetics, we conducted additional simulations in USP Apparatus II of drug particles with different dissolution rate constants. To accommodate computational demand, we used a lower-resolution 101 × 101 × 121 grid to conduct the simulation. Figure 19 shows the concentration profiles. Not surprisingly, a higher intrinsic dissolution constant leads to faster dissolution in the vessel (where the paddle speed is kept the same).

### Simulation Model Performance

**Fig. 20:**
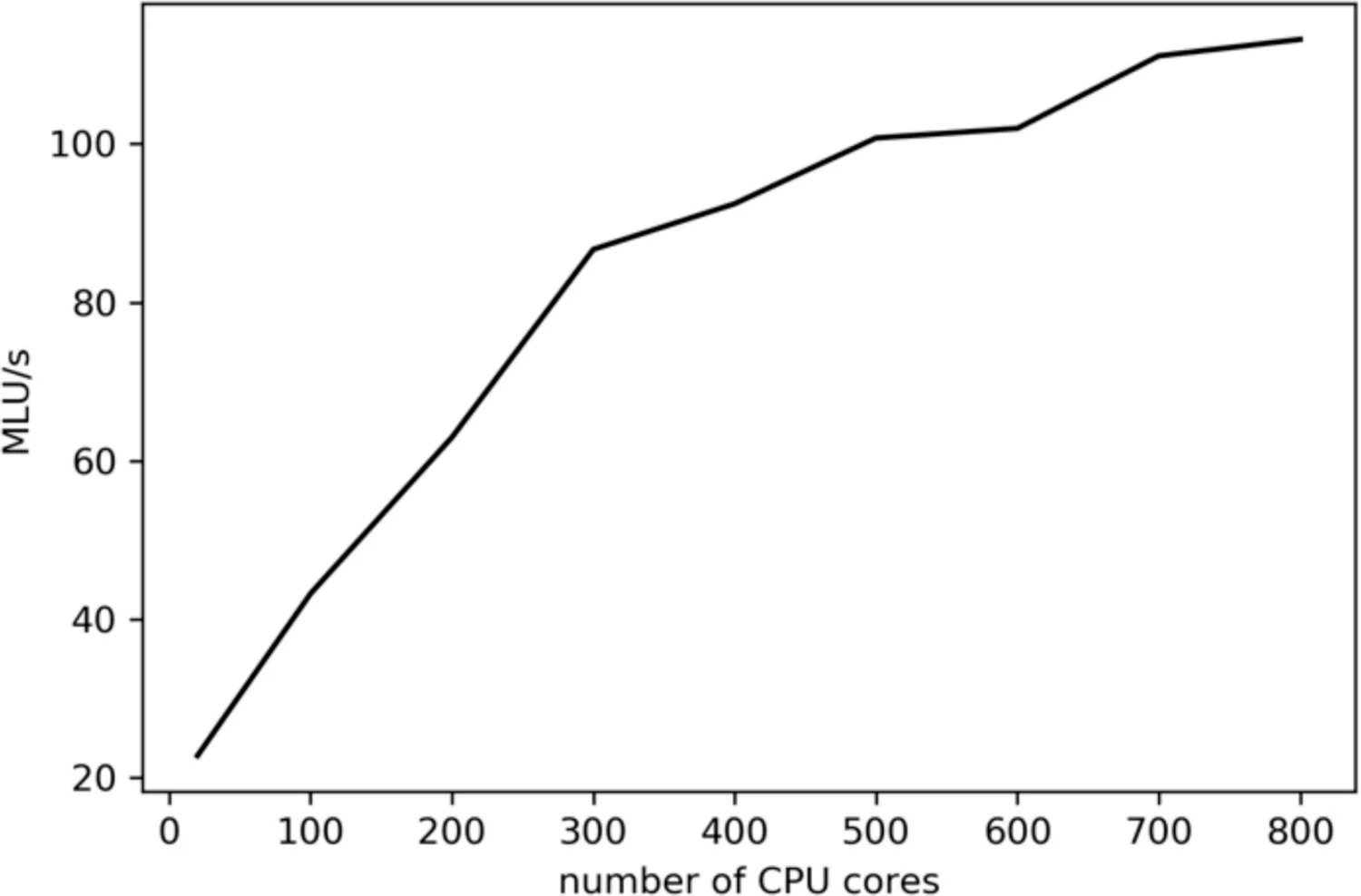
Scalability is indicated by millions of lattice units processed per second as a function of the number of CPU cores.

The coupled LBM and DEM simulations were conducted on a Linux cluster with multiple nodes and cores. The cluster consists of HP compute nodes, each equipped with two 10-core Intel Xeon E5 processors (totaling 20 cores per node) and 128 GB of memory, interconnected via 100 Gbps EDR InfiniBand. Each simulation lasted at least 1 h to ensure stable data collection. The LBM component was the performance-limiting step, while the DEM of particle dynamics was considerably faster. Figure 20 analyzes and displays the scaling of the LBM fluid dynamics computation. The LBM speed increased roughly linearly as the number of cores reached 300. Further increases in core count resulted in slower performance gains, likely due to communication overhead between the LBM and DEM. For immediate-release cases, this runtime is practical for mechanistic studies, but it could become burdensome for controlled-release profiles extending over many hours if the same full-resolution approach were used.

Expanding the framework to controlled-release tablets is theoretically possible, but it will need faster algorithms, such as adaptive or multi-rate time stepping, coarse-grained particle models, reduced-order transport updates during slow periods, and additional parallel optimization. We state this clearly to show that the current work introduces the coupled framework and proof of concept for immediate-release, rather than a ready long-term simulator.

The simulation conducted in this study demonstrated that coupled LBM and DEM could simulate multiple particles moving in an agitated fluid environment. Simulation results reproduced the experimental hydrodynamic results from the USP Apparatus II, illustrating the suitability and fidelity of LBM for computing fluid dynamics at the scale of a dissolution device used in the pharmaceutical industry. Additionally, we successfully implemented the coupling between LBM and DEM, as demonstrated by the coordinated kinetics between fluid and particle dynamics. This coupling will further allow us to integrate particle dissolution in the computational framework and simulate tablet disintegration and dissolution.

## Conclusion

Prediction of tablet dissolution based on tablet microstructure, formulation, and manufacturing factors is challenging. This paper demonstrates the development of coupled LBM-DEM methods to simulate particle dissolution in the USP Apparatus II. Two-way coupling between solid particles and an aqueous fluid allows real-time simulations of particle dissolution kinetics using a single open-source platform. Our model leverages LBM’s scalability, the ease of implementing fluid dynamics, and DEM’s flexibility to compute individual particle motion, collisions, and interactions. The immersed moving boundary scheme was implemented to fully account for the hydrodynamic conditions caused by paddle rotation in the mass transfer and convection–diffusion kinetics of drug particles. Our computational framework also enabled the simulation of tablet compression and the creation of a model tablet for dissolution testing. The results show that coupling LBM and DEM can effectively simulate dissolution in the USP Apparatus II. Our research further demonstrates the possibility of incorporating particle swelling, granule breakage, and other kinetic events into the simulation framework, supporting continuous improvement. However, this study has several key limitations: the tablet-generation process is simplified; excipients are not modeled explicitly; validation with a single particle is qualitative; and controlled-release timescales are still computationally intensive. These limitations guide future model development but do not detract from the main achievement: establishing a stable, two-way coupled hydrodynamic-particle-dissolution framework.

From a practical perspective, the proposed framework provides a scalable, mechanistic tool for analyzing tablet dissolution under compendial hydrodynamic conditions and for interpreting dissolution behavior at the full-vessel scale. While high-resolution micromechanical models (e.g., bonded multi-sphere and multi-contact DEM approaches) offer rich particle-level insight into the mechanical tableting process [65, 73], their computational expense often precludes simulation of fluid-driven dissolution over the spatial and temporal scales relevant to standard dissolution tests. The proposed LBM–DEM framework is therefore positioned as a deliberate, industry-relevant balance between mechanical fidelity and computational scalability: it simplifies the initial mechanical state to remain tractable, while strictly resolving the two-way coupling among fluid flow, mass transfer, and particle-scale dissolution/redistribution. This two-way coupling is not merely a mathematical feature; it is the mechanism that enables the model to reproduce emergent macroscopic phenomena, such as localized flow impedance, the coning effect, and persistent spatial concentration gradients, that can dominate observed dissolution outcomes yet are systematically missed by one-way or spatially averaged descriptions. Pragmatically, this capability helps practitioners distinguish apparatus-induced hydrodynamic artifacts from intrinsic formulation limitations, supporting diagnosis of dissolution variability and root-cause analysis in out-of-specification (OOS) investigations. As a predictive complement to physical experiments (rather than a replacement), the framework further enables efficient “what-if” evaluation of operating conditions, tablet structure, and particle-size distributions within a Quality by Design (QbD) paradigm, thereby focusing confirmatory testing, accelerating design space exploration, and informing process optimization.

## Data Availability

The simulation data, input scripts and post-processing utilities supporting the findings of this study are available from the corresponding author upon request.
